# High-throughput screening of multifunctional nanocoatings based on combinations of polyphenols and catecholamines

**DOI:** 10.1016/j.mtbio.2021.100108

**Published:** 2021-03-10

**Authors:** F. Behboodi-Sadabad, S. Li, W. Lei, Y. Liu, T. Sommer, P. Friederich, C. Sobek, P.B. Messersmith, P.A. Levkin

**Affiliations:** a Institute of Biological and Chemical Systems – Functional Molecular Systems (IBCS-FMS), Karlsruhe Institute of Technology (KIT), Eggenstein-Leopoldshafen, 76344, Germany; b Institute of Theoretical Informatics, Karlsruhe Institute of Technology (KIT), Am Fasanengarten 5, Karlsruhe, 76131, Germany; c Institute of Nanotechnology, Karlsruhe Institute of Technology (KIT), Hermann-von-Helmholtz-Platz 1, Eggenstein-Leopoldshafen, 76344, Germany; d Departments of Bioengineering and Materials Science and Engineering, University of California Berkeley, CA, 94720-1760, USA; e Materials Sciences Division, Lawrence Berkeley National Laboratory, Berkeley, CA, 94720, USA

**Keywords:** Multifunctional nanocoating, Plant polyphenol, Catecholamine, Miniaturized platform, High-throughput screening

## Abstract

Biomimetic surface coatings based on plant polyphenols and catecholamines have been used broadly in a variety of applications. However, the lack of a rational cost-effective platform for screening these coatings and their properties limits the true potential of these functional materials to be unleashed. Here, we investigated the oxidation behavior and coating formation ability of a library consisting of 45 phenolic compounds and catecholamines. UV–vis spectroscopy demonstrated significant acceleration of oxidation and polymerization under UV irradiation. We discovered that several binary mixtures resulted in non-additive behavior (synergistic or antagonistic effect) yielding much thicker or thinner coatings than individual compounds measured by ellipsometry. To investigate the properties of coatings derived from new combinations, we used a miniaturized high-throughput strategy to screen 2,532 spots coated with single, binary, and ternary combinations of coating precursors in one run. We evaluated the use of machine learning models to learn the relation between the chemical structure of the precursors and the thickness of the nanocoatings. Formation and stability of nanocoatings were investigated in a high-throughput manner via discontinuous dewetting. 30 stable combinations (hits) were used to tune the surface wettability and to form water droplet microarray and spot size gradients of water droplets on the coated surface. No toxicity was observed against eukaryotic HeLa cells and *Pseudomonas aeruginosa* (strain PA30) bacteria after 24 h incubation at 37 °C. The strategy introduced here for high-throughput screening of nanocoatings derived from combinations of coating precursors enables the discovery of new functional materials for various applications in science and technology in a cost-effective miniaturized manner.

## Graphical abstract

**Figure:**
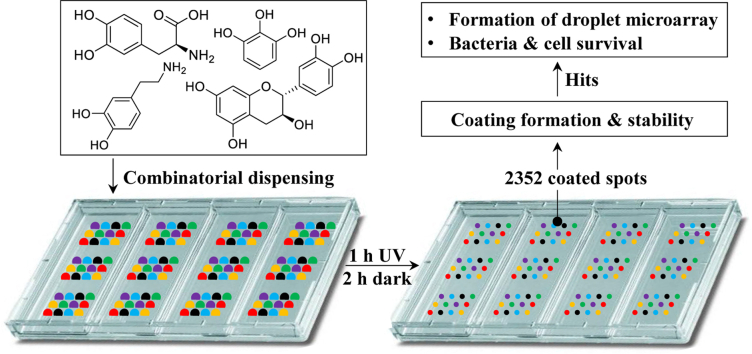

## 1 Introduction

Dopamine and other catecholamines have been widely used as a versatile material-independent surface functionalization toolbox [1] with numerous applications in tissue engineering, drug delivery, photothermal therapy, water treatment, membranes, energy, and gas separation [2]. Another attractive group of materials used for surface functionalization and material engineering is the large class of plant polyphenols [3]. Polyphenols have gained enormous consideration thanks to their interesting physicochemical peripeties, important roles in plants, vast presence in the human diet, and their antioxidant and anticancer properties [3,4].

Functional nanocoatings derived from plant polyphenols (such as tannic acid) and catecholamines (such as dopamine) have been widely used to develop advanced functional materials for various applications [2,3,5]. One of the main advantages of this method is the simplicity with which different materials can be functionalized. A substrate is typically immersed into a solution of a plant-derived phenolic compound or a catecholamine to form a nanometer-thin coating on the surface. Polymerization and deposition of both polyphenols and catecholamines usually require one of the following: slightly basic conditions [[6], [7], [8], [9], [10], [11]], presence of an oxidizing agent [[12], [13], [14]], metal ion complexation [15,16], microwave [17], or UV [6,8,18] irradiation.

Functional and physicochemical properties of catecholamine-based nanocoatings have been improved by using a diversity of building blocks of catecholamines such as dopamine [11], norepinephrine [19], 3,4-dihydroxy-l-phenylalanine (DOPA), and their derivatives [1,20]. The enormous biological diversity of plant polyphenols with a broad range of biological and chemical properties is important for the development of novel functional coatings [1,5]. In one of the first reports on using plant polyphenols to form substrate independent nanocoatings, Sileika et al. [9] demonstrated the formation of functional nanocoatings on a variety of substrates via autoxidation of pyrogallol, tannic acid, pure plant polyphenols, including epigallocatechin gallate (EGCG), epicatechin gallate (ECG), and epigallocatechin (EGC), and crude extracts of polyphenol-rich foods (such as red wine and green tea). The coatings were expanded to 15 natural polyphenols later [10].

Several factors affect the deposition behavior and the final properties of the coatings derived from plant polyphenols, catecholamines, and their derivatives. For example, the effect of buffer pH, ionic strength, or choice of the solvent of the precursor solution, type of oxidant and oxidation conditions, the concentration of the precursor, influence of UV light on polymerization and deposition have been demonstrated [2,20]. The presence of such a high number of variables together with a broad diversity of available starting compounds requires high-throughput methods to unleash the vast potential of these functional coatings. Most studies, however, focused on one-by-one or low-throughput evaluations utilizing either single catecholamine [1,2] or a phenolic compound [9,10] as precursors. For example, Jeon et al. [21] investigated coating formation from four binary combinations of plant phenolic compounds (including catechin-catechol, catechin-syringic acid, catechin-tannic acid, ferulic acid-catechol) obtained by enzyme induced polymerization. They observed a boost in the coating deposition speed for these four binary combinations, which was attributed to the contribution of the non-catechol structures to the surface adhesion and increase of the polymerization extent via the hetero-coupling process [21]. Several other methods have been developed to enhance the functional properties of the nanocoatings by combining the primary catecholamine with a second compound to make a binary precursor such as copolymerization of dopamine or DOPA with a functionalized dopamine analog, and co-deposition with nucleophilic cross-linkers [2,20].

High throughput methods have been used to screen a variety of coating precursors and their combinations to investigate their processing conditions and final properties [22]. For instance, Ana l. Neto et al. [23] reported a high throughput screening strategy to form and characterize multilayer films using a layer-by-layer methodology. They studied mechanical properties and cell adhesion of the cell thin films made of dopamine-modified hyaluronic acid. Hou et al. [24] developed a bicomponent polymer pattern by coating polydopamine (PDA) on super-hydrophilic polymer brushes, which was used for protein and cell studies. In a recent study, Guo et al. [25] used polydopamine (PDA) coated polydimethylsiloxane (PDMS) nanosubstrates to prepare a platform for rapid cell-pattering with potential application in high throughput cell studies. They prepared the PDA-patterned surface using a lift-off lithography process in four-step.

In this work, we developed a miniaturized platform that enables the rapid formation and high-throughput screening of nanocoatings. A library of structurally diverse phenolic compounds and catecholamines was used to form coatings, which were investigated by UV–vis spectroscopy, ellipsometry analysis, and wettability measurements. Using single, binary, and ternary combinations of precursors, an array of 2,532 spots with different compositions of polyphenolic coatings were studied in a high-throughput manner. 30 identified stable nanocoatings were utilized to create hydrophilic-hydrophobic microarrays by direct liquid deposition onto a polystyrene Petri-dish in order to form droplet microarrays using the effect of discontinuous dewetting. The toxicity and biocompatibility of the nanocoatings were also investigated.

## 2 Materials and methods

### 2.1 Materials

Chemical compounds 3-nitrophenol (**P3**), (−)-epigallocatechin gallate hydrate (**P41**), naringin hydrate (**P43**) were purchased from Tokyo Chemical Industry (America). Caffeic acid methyl ester (P20) was purchased from abcr GmbH (Germany). All the other chemicals were purchased from Sigma-Aldrich. Chemicals were used without further purification. High-purity DI water with a resistivity of 18.2 MΩ cm was obtained from an in-line Millipore water purification system. Tris buffers were made at 10 mmol/L concentration at pH 8.5. The final pH value was adjusted by using a METTLER TOLEDO digital pH meter. Acetone and the other solvents were obtained from Merck KGaA.

Falcon polypropylene conical tubes and 96-well UV-Transparent Microplates were purchased from Corning (Corning, US). The combinatorial coating precursors were dispensed on non-treated rectangular 4-well cell culture plates (Thermo Scientific™, Nunc™) in high-throughput experiments. Nexterion B glass slides obtained from Schott AG and silicon wafers (CZ-Si-wafer 4 in.) from MicroChem GmbH were used. Silicon wafers and silicon wafers with a layer of 100 nm titanium oxide (TiO_2_) were obtained from University Wafer, Inc (Boston, MA). TiO_2_-coated silicon wafers and silicon wafers were cut into 1 cm × 1 cm pieces and subsequently cleaned by sonication in the following media: DI water, 2-propanol, and ethanol for 20 min each and dried with nitrogen gas. A non-treated sterile 4-well dish (127.8 × 85.5 mm, Thermo Fisher Scientific, USA) was used as the target substrate for coating as received. UV plate with UV transparent Flat bottom (acrylic, 96 well, Corning, USA) was used for UV–vis spectroscopy.

Hoechst 33342 (1.0 mg/mL in water), Calcein AM (10 mg/mL in DMSO) and propidium iodide (PI, 1.0 mg/mL in water) were purchased from Thermo Fisher Scientific Inc. (MA, USA). Fetal calf serum (FCS), Dulbecco's Modified Eagle's Medium (DMEM), 0.25% trypsin/EDTA, and 1% penicillin/streptomycin solution were purchased from Gibco, Life Technologies GmbH (Darmstadt, Germany).

### 2.2 Deposition of nanocoatings

#### 2.2.1 Inside the falcon tubes

A precursor solution (1 mg/mL) of the soluble compounds was prepared in tris buffer (10 mM Tris buffer, pH 8.5). 5 mL of each solution was transferred into a 15 mL falcon tube and sealed properly. The solutions were stored in a falcon tube (blue cap on top) in dark for 48 h. Photographs of the solutions were taken at 30 min, 6 h, 12 h, 24 h, and 48 h time intervals, while the tube was turned upside down to move the solution away from the coated area (the blue cap at the bottom) for photography. The falcon tubes were stored as before in dark (blue cab on top) after taking the photographs.

#### 2.2.2 On TiO_2_ surface for ellipsometry

Cleaned substrates were immersed in tris buffer (1 mg/mL, 10 mM Tris buffer, pH 8.5) and were irradiated with UV light (320–450 nm, 400 Watt, metal halide bulb, Dymax, Model 2000 Flood) for 1 h followed by 2 h incubation in dark environment. Modified substrates were then rinsed thoroughly with DI water and ethanol and dried with nitrogen. The same conditions were used to make a coating layer on the silicon surface under UV irradiation for 30 min.

#### 2.2.3 Using a non-contact liquid dispenser

Precursor solutions (1 mg/mL, 10 mM Tris buffer, pH 8.5) of the individual compounds were prepared and were fed into the source plate of a non-contact liquid dispensing device (Immediate Drop On-Demand technology (I-DOT) liquid dispenser; Dispendix, Stuttgart, Germany). A customized program was used to print desired combinations of the precursors on a predefined spot on the surface of a 4-well plate (target substate) with a controlled order of printing of the components in an environment with controlled humidity (80%). After printing arrays of precursor solutions on the surface, the substrate was transformed into a UV chamber (365 nm, 5 mW/cm^2^) and was irradiated for 1 h followed by storage in dark for 2 h. A humid environment was created by placing wet humidifying pads around the substrate to prevent evaporation of the droplets. The substrate was washed with water and ethanol thoroughly several times after coating deposition.

### 2.3 Stability of the nanocoatings

A 4-well plate was coated with the 588 combinations using the I-DOT dispenser as explained before. After 24 h, the coated substrate was immersed in water followed by tilting to spontaneously form water droplet microarrays on the coated spots due to the contrast of wettability. Images of the droplets formed on the surface were taken using a bright field microscope (Modellreihe BZ-9000) and merged. Another coated substrate was prepared under the same condition but was immersed in ethanol for 24 h. The substrate was immersed in water and tilted to form a droplet microarray on the coated spots derived by the contrast of wettability afterward followed by imaging as explained. The size of the droplets was determined by ImageJ software and an average was obtained from four times repetition of each experiment.

### 2.4 Formation of droplet microarray and spot size gradients of droplet microarrays

Precursor solutions of the hits were prepared and fed into the source plate of the I-DOT dispenser as explained before. The precursor solution of the combinations was printed on predefined spots on the surface with 5 times repetitions. The substrate was washed and dried and then was immersed in ethanol for 24 h. The coated substrate was immersed in water and tilted in order to form a droplet microarray (DMA) on the surface derived by the contrast of the wettability. In order to form spot size gradients of the DMA on the surface, the printed volume of the precursor solution was varied from 30 nL to 210 nL on predefined spots on the surface, followed by coating deposition (1 h UV irradiation and 2 h incubation in dark), washing, and treatment with ethanol for 24 h, as explained before. A spot size gradient of water droplets with diameters from 150 μm to 1,000 μm was spontaneously formed on the surface following the immersing of the coated substrate in water and tilting it.

### 2.5 Characterization

#### 2.5.1 UV–vis spectroscopy

UV–vis spectroscopy was performed with an Infinite M200 Pro (Tecan Trading AG, Switzerland) plate reader. Stock solutions of the precursors were prepared (1 mg/mL, 10 mM Tris buffer, pH 8.5) and stored in dark. In order to prepare the 1:1 (volume ratio) solution of two compounds, a stock solution of each compound was prepared and mixed with a 1:1 volume ratio. For measuring the UV–vis absorbance at each time interval, 200 μL of the stock solution was transferred to a UV transparent well plate and fed into the plate reader. The UV–vis absorbance of the precursor solutions (buffer as the reference) was measured at different time points (0 min, 30 min, 60 min, 90 min, 120 min, 6 h, 12 h, and 24 h for the dark samples. For the UV irradiated sample UV-absorbance was recorded after 15 min UV irradiation.

#### 2.5.2 Ellipsometry

The thickness of the deposited layer on the TiO_2_ substrates was measured with a spectroscopic ellipsometer (M-2000V, J.A. Woollam, USA). The spectra were fitted with multilayer slab models using CompleteEase software. The TiO_2_ layer was fitted with a Cauchy model immediately before immersion in the precursor solution and the thickness of the deposited layer on the TiO_2_ was fitted with a B-spline model. An average thickness was obtained from three independent experiments. The coefficients of the Cauchy model were fixed to An = 2.02 and Bn = 0.21 to fit the TiO_2_ layer. In the B-spline model used to fit the deposited layer, the n and k values were set to initial values of 1.5 and 0.0 respectively. The variable angle spectroscopic ellipsometric (VASE) data (Psi, Delta) in the spectral region of 370–900 nm were obtained at the angle of incidence of 65°. In another set of measurements, VASE was performed at angles of incidence of 55°, 60° in addition to 65°, and the initial n and k values were allowed to be fitted. No statistically significant variation in the calculated thickness values was obtained neither at different angles of incidence used nor by fitting the initial values of n and k.

### 2.6 Antimicrobial properties

The bacteria used in this study is *Pseudomonas aeruginosa* strain PA30 [26]. The 96-well plate with the nanocoatings on the bottom were sterilized by pipetting 200 μL 70% ethanol and incubating for 10 min. *P. aeruginosa* PA30 were inoculated in basal medium 2 (BM2; 62 × 10^−3^ M potassium phosphate, 7 × 10^−3^ M (NH_4_)_2_SO_4_, 2 × 10^−3^ M MgSO_4_, 10 × 10^−6^ M FeSO_4_, and 0.4% glucose) [27] and incubated at 37 °C with 100 rpm shaking overnight. Then bacterial overnight culture solution was adjusted to an optical density of 600 nm (OD600) of 0.1 (≈1 × 10^8^ bacteria per mL) and 200 μL of the bacterial solution was inoculated into each well of the 96-well plate with the nanocoatings on the bottom. The 96-well plate containing coating precursor compounds and bacterial solution were incubated for 24 h at 37 °C statically. Bacterial solution was removed from the 96-well plate after the incubation and each well was rinsed with cell wash buffer (5 × 10^−3^ M magnesium acetate, 10 × 10^−3^ M Tris-base, pH 8) for three times (200 μL buffer for each well per time). Then 200 μL 5-Cyano-2,3-ditolyl tetrazolium chloride (CTC, from Polysciences Europe GmbH (Eppelheim, Germany), 4 × 10^−3^ M) BM2 solution was added into each well and incubated for 3 h at 37 °C. CTC solution was removed from wells after the incubation and each well was rinsed three times with cell wash buffer. 200 μL of 4’,6-diamidino-2-phenylindole (DAPI, 1 μg/mL) water solution was added into each well and incubated at room temperature for 10 min. Then DAPI solution was removed from the wells. Each well was rinsed three times with buffer. AxioImage M2 system equipped with an Apotome (Carl Zeiss, Oberkochen, Germany) was used to image the stained samples. At least three repetitions were prepared for each sample. The fluorescence intensity of stained bacteria was calculated by ImageJ. All the values were normalized with the fluorescence intensity of stained bacteria on bare PS surfaces. Fluorescence of CTC staining represents alive bacteria, while the fluorescence of DAPI staining represents all bacteria (live and dead) [28].

### 2.7 Cell toxicity

Human cervical adenocarcinoma HeLa cell line (HeLa CCL2) was obtained from DSMZ GmbH (Leibniz Institute DSMZ German collection of microorganisms and cell cultures). HeLa cells were trypsinized and suspended with DMEM medium containing 10% FCS and 1% penicillin/streptomycin till the cell concentration reaches 1.0 × 10^5^ cells/mL. Then 100 μL cell suspension was added into the individual wells of the 96-well plate, which were already coated with different combinations of compounds. Cells were incubated in a 37 °C incubator, then 10 μL per well Hoechst 33342 (1 μg/mL), Calcein AM (0.5 μg/mL) and PI (0.5 μg/mL) solution was added into each well and treated cells for 15 min. Four images of the individual wells were taken using Olympus IX81 inverted motorized microscope (Olympus, Tokyo, Japan) with 6.4× magnification. The number of Hoechst 33342- and PI-positive cells were counted using ImageJ (http://imagej.nih.gov/ij/). Cell viability was calculated as the following equation: cell viability (%) = (1-PI positive cell numbers/Hoechst 33342 positive cell numbers) × 100.

## 3 Results and discussion

**Fig. 1:**
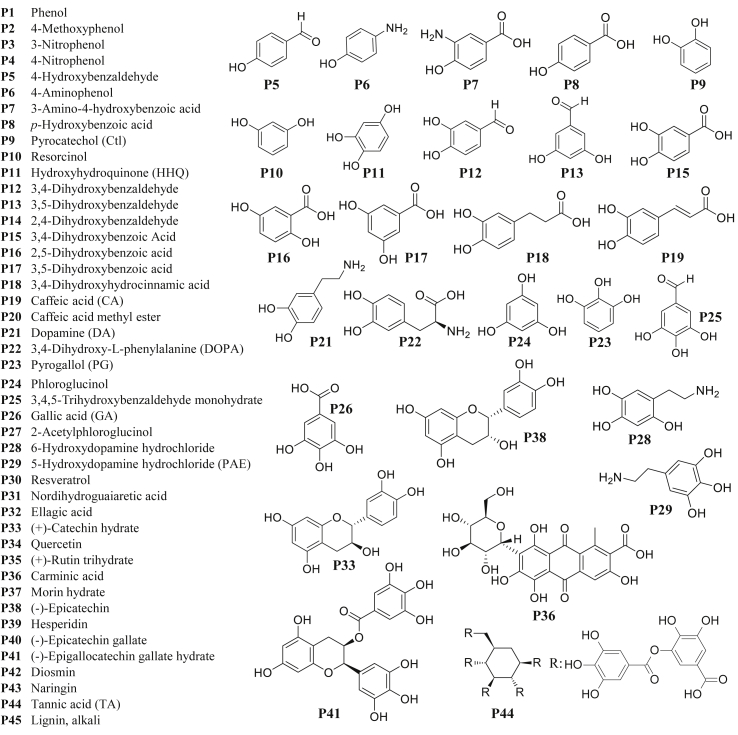
Chemical compounds (phenols and catecholamines) investigated in this study (left). Selected chemical structures are shown on the right (for all structures see Fig. S1).

In our previous reports [6,8,18,29], we demonstrated the use of basic pH conditions and UV irradiation to induce oxidation and polymerization in catechol and pyrogallol-containing compounds. It is known [30,31] that oxidation of catechol and pyrogallol-containing compounds leads to the formation of quinone intermediates (with the emergence of an absorbance peak at around 390 nm) and subsequent cross-linking or cyclization leading to phenol-coupled polymer products (with increased absorbance around 450 nm) [32]. Here, in order to investigate the oxidation behavior of selected compounds, we analyzed the single component precursor solutions (1 mg/mL, 10 mM Tris buffer, pH 8.5) (Fig. 1, Fig. S1) by UV–vis spectroscopy in dark over 24 h or after 15 min UV irradiation (Fig. S2–Fig. S12). We observed that the oxidation behavior of the 36 soluble (out of the total 45) compounds in the given conditions was dependent on multiple factors, including the number and distribution of phenolic OH groups and the presence of additional functional groups, especially amines. For instance, the UV absorbance monitored between 300 and 700 nm was not increased significantly in basic pH for the compounds having one phenolic OH group without further functional groups (**P1**) or with methoxy (**P2**), nitro (**P3**, **P4**), aldehyde (**P5**), or carboxylic acid (**P8**) functional groups directly on the benzene ring. However, a relatively high increase in the UV absorbance was observed for **P6** and **P7** that contain one primary amine directly attached to the benzene ring (Figs. S2–S3).

For compounds having two phenolic OH groups (**P9**, **P10**) with different further chemical functional groups, including aldehyde (**P12**, **P13**), carboxylic acid (**P15**, **P18**, **P19**), ester (**P20**), amine (**P21**), or both amine and carboxylic acid (**P22**), the UV absorbance was significantly increased in basic pH or under UV irradiation (Figs. S4–S6). The increase in the UV absorbance of **P14** (with aldehyde group), **P16**, and **P17** (with the carboxylic acid group) were relatively low in dark, dissimilar to the rest of the compounds in this category. However, a stronger increase in the UV absorbance was observed for these three compounds after UV irradiation compared to the absorbance of the samples stored in dark, indicating the strong influence of UV on the oxidation and polymerization of these phenolic compounds (Figs. S5–S6). Amine-containing compounds (**P21**, **P22**) in this group showed a strong increase of the UV absorbance both in dark and under UV irradiation (Fig. S7).

Compounds with three phenolic OH groups in which at least two OH groups were in ortho position with respect to each other showed a drastic increase of the UV absorbance in a broad range of wavelengths for samples stored in dark or after only 15 min UV irradiation (Figs. S7–S8). This behavior was observed for the compounds without additional functional groups (**P11**, **P23**) and for the compounds with one aldehyde (**P25**), carboxylic acid (**P26**), or amine (**P28**, **P29**) groups. However, UV absorbance of the compounds lacking adjacent phenolic OH groups, including **P24** and **P27** was not increased in the dark environment, while UV absorbance was increased after 15 min of UV irradiation (Figs. S7–S9). Some of the higher molecular weight compounds, including **P30**, **P31**, **P32**, **P34**, **P35**, **P39**, **P42**, **P43**, **P45** were not soluble in the same given conditions and were not investigated. A relatively high increase in the UV absorbance for the samples stored in dark or under UV irradiation was observed for compounds with multiple phenolic OH groups in which the OH groups were distributed on more than one benzene ring (**P33**, **P38**, **P40**, **P41**, **P44**) (Figs. S10–S12). Exceptions in this category that showed a very low change in the UV absorbance were **P36** and **P37**.

It should be noted that in 32 out of the 36 soluble compounds, 15 min of UV irradiation of their solutions significantly accelerated the oxidation and possible polymerization of these compounds as evidenced by the observed increase of absorbance in the UV–Vis region. This phenomenon was previously shown for dopamine [18,29] and some phenolic compounds, including tannic acid, caffeic acid, pyrogallol, gallic acid, pyrocatechol, catechin, and epigallocatechin gallate [6,8].

**Fig. 2:**
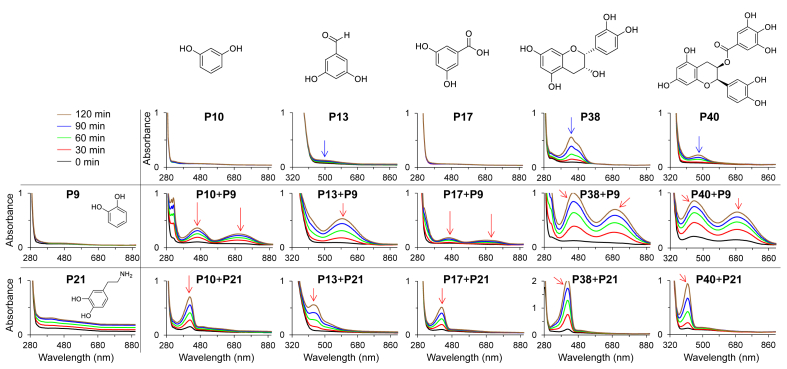
Oxidation behavior of 10 selected binary combinations of compounds (for the other 12 combinations see Fig. S13). UV–vis spectra of precursor solution of the combination of **P9** (pyrocatechol) and **P21** (dopamine) with five other compounds (**P10**, **P13**, **P17**, **P38**, **P40**) measured in 30 min time intervals. The appearance of new peaks (marked with red arrows) and alteration of the intensity of the characteristic peaks of the individual compounds (marked with blue arrows) in the precursor solution indicates a possible different oxidation pathway and oxidation kinetics for the combinations compared to the individual compounds.

It has been shown that the presence of both catechol (such as in **P9**: pyrocatechol) and primary amino (such as in **P21**: dopamine) groups are advantageous for the formation of robust nanocoatings [1,2,20]. Therefore, **P9** and **P21**, which are among the most commonly used building blocks in catecholamine-based materials, were selected to investigate the oxidation and deposition of nanocoatings derived from combinations. By recording UV–Vis spectra we investigated the oxidation behavior of binary combinations composed of **P9** (pyrocatechol) or **P21** (dopamine) as one component and one of the following second phenolic compounds (Fig. 2, Fig. S13): **P10**, **P13**, **P17**, **P38**, **P40** (Fig. 2), **P14**, **P12**, **P24**, **P27**, **P26**, **P33** (Fig. S13). UV–Vis spectra of the combinations showed new peaks not existing in the spectra of the individual compounds, with an exception to **P14** and **P12** where no new peak was observed even after 120 min incubation in dark. The appearance of new peaks around 425 nm during autoxidation of dopamine in the presence of the phenolic compounds having two non-adjacent phenolic OH groups in one ring might be attributed to the formation of monardine and azamonardine adducts [[33], [34], [35]]. Amin et al. [35] identified adducts of dopamine and resveratrol and proposed that following electrophilic substitution of resveratrol and additional oxidation of the DA fragment, cyclization by Michael addition of a pendant phenol of the resveratrol fragment onto the DA carbon bearing the aminoethyl moiety can occur [35]. It was proposed that the azamonardine adducts were formed via a nucleophilic addition reaction followed by a tautomerization [35]. The alteration of the intensity of the peaks originated from individual compounds (marked with blue arrows) and the appearance of new peaks (marked with red arrows) in the UV–vis spectra of the combinations (Fig. 2) suggest that a different oxidation pathway occurred in the combinations compared to the individual compounds. These differences could lead to the formation and deposition of surface coatings of unique composition. A detailed mechanistic study will be required to characterize the oxidation and polymerization pathways of these reactions.

**Fig. 3:**
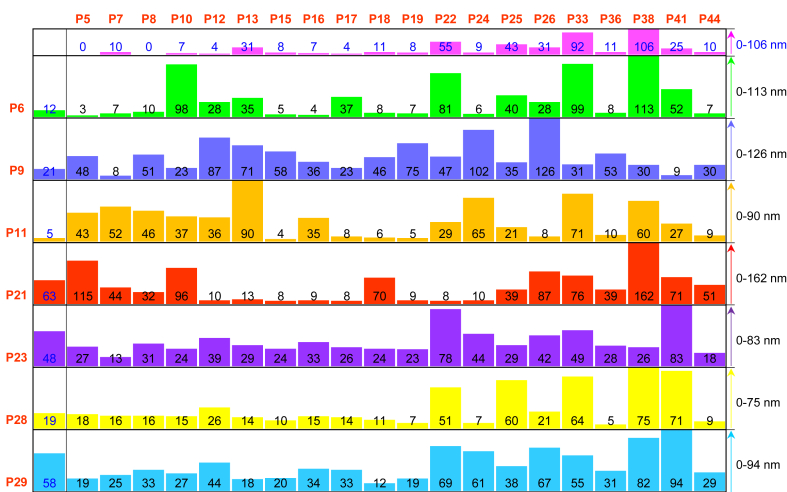
Thicknesses (nm) of nanocoatings derived from 27 selected phenolic compounds (in red font color) and some of their 1:1 binary combinations (1:1 volume ratio, each component at 1 mg/mL, 10 mM Tris buffer, pH 8.5), measured on titanium dioxide surface after 1 h UV irradiation, followed by 2 h incubation in dark. Thicknesses (nm) of the nanocoatings derived from single component precursor solutions are shown in the first column and first row (in blue font color). Thicknesses (nm) of nanocoatings derived from each binary mixture can be found at the intersections of the corresponding columns and rows. The height of the columns in each row is normalized to the maximum thickness value in the same row. Maximum and minimum thickness values in each row are shown on the right side. Thickness values (nm) with error bars are shown in Fig. S15.

Having analyzed the oxidation behavior of the individual compounds and some of their binary combinations, we investigated the ability of these combinations to form coatings. 27 soluble compounds that demonstrated a significant increase in the UV absorbance after 15 min UV irradiation compared to dark conditions were selected in a manner to include specific and diverse functional groups (for chemical structures see Fig. 1) to investigate their coatings, either individually or in combinations. First, precursor solutions of the individual compounds were prepared (1 mg/mL, 10 mM Tris buffer, pH 8.5) and stored in dark. Then, photographs of these solutions were taken in several time intervals at 30 min, 6 h, 12 h, 24 h, and 48 h after the preparation (Fig. S14). A visible dark layer was formed on the inner surface of the falcon tubes from the precursor solutions of the phenolic compounds **P6**, **P9**, **P13**, **P21**, **P22**, **P23**, **P25**, **P26**, **P28**, **P33**, and **P38** (Fig. S14). The thickness of the nanocoatings derived from these selected 27 compounds and thickness of the nanocoatings derived from selected 1:1 mixtures of binary precursor solutions (1:1 vol ratio, each component at 1 mg/mL, 10 mM Tris buffer, pH 8.5) were measured by ellipsometry on a titanium dioxide surface after 1 h UV irradiation followed by 2 h incubation in dark (Fig. 3 and Fig. S15: column graph representation of the same data with error bars). Under the same coating conditions, we observed that the thickness values of the nanocoatings were dependent on the type and composition of the precursors (chemical structure and presence of specific functional groups) (Fig. 3). Table S1 summarizes oxidation behavior and the thickness values of the individual compounds. Following is a summary of our observations of the relationship between film thicknesses and the structure of phenolic compounds (Fig. 3A):

- Compounds with one phenolic OH group and one amino group, **P6**, and **P7**, gave coatings of 12 ± 3 nm and 10 ± 2 nm, respectively, while the two other compounds with a single OH group but not amino group did not yield a nanocoating (**P5** and **P8**). **P6** and **P7** demonstrated a high increase in the UV absorbance, while that of **P5** and **P8** was negligible (Fig. S3).
- **P9** possessing two adjacent OH groups (ortho position) yielded a 21 ± 6 nm coating whereas **P10** possessing two non-adjacent OH groups in para position gave a 7 ± 3 nm coating. Both compounds demonstrated an increase in the UV absorbance in dark and under UV irradiation. For compounds with two phenolic OH groups and one aldehyde group (**P12**, **P13**), the highest increase in the UV absorbance (Fig. S5) and the highest coating thickness (31 ± 4 nm) were obtained for the isomer having two OH groups in meta position (**P13**). Compounds having two phenolic OH groups and one carboxylic acid (**P15**, **P16**, **P17**) demonstrated a relatively low increase in the UV absorbance for the samples stored in dark (Fig. S6). Although UV irradiation could increase the UV absorbance, the thickness of the nanocoatings was low (8 ± 1, 7 ± 2, and 4 ± 1 nm, respectively). Connecting carboxylic acid to catechol via an alkyl or alkylidene bridge (**P18**, **P19**) increases coating thickness to 11 ± 2 and 8 ± 2 nm, respectively. **P21**, **P22** (compounds with two adjacent OH groups containing one amino group) resulted in high thicknesses of 63 ± 5 nm and 55 ± 7 nm, respectively, demonstrating also relatively high UV absorbance of solutions both in dark and under UV irradiation (Fig. S7).
- A significantly higher thickness (48 ± 4 nm) was obtained for pyrogallol (**P23**) compared to its constitutional isomer phloroglucinol (**P24**) (9 ± 2 nm). An increase in the UV absorbance of pyrogallol was observed in a wide range of wavelengths (300 nm–700 nm) even in the dark, while **P24** led to a change in the UV absorbance above 300 nm only after UV irradiation (Fig. S8). Similar behavior in the oxidation of the precursor and deposition of the nanocoating was observed for **P27** (Fig. S8). Hydroxyhydroquinone **P11** demonstrated a high increase in the UV absorbance in dark and under UV irradiation (Fig. S4). However, the thickness of the corresponding nanocoating was the lowest (5 ± 1 nm) among the compounds with three phenolic OH groups.
- Relatively high UV absorbance in both dark and under UV irradiation and thick coatings were obtained for the rest of the compounds with three phenolic OH groups possessing additional functional groups such as one aldehyde **P25** (43 ± 5 nm), or one carboxylic acid **P26** (31 ± 4 nm), or one amino group **P28** (19 ± 2 nm) and **P29** (58 ± 5 nm). **P36** resulted in low coating thickness (11 ± 2 nm), and the UV absorbance was increased only after UV irradiation. In spite of relatively high increase in the UV absorbance of **P44** solution, the corresponding coating was only 10 ± 2 nm.
- Among compounds with phenolic OH groups distributed on multiple rings, the thickest coatings were obtained for **P33** (92 ± 7 nm) and **P38** (106 ± 9 nm), possessing one aromatic ring with two adjacent phenolic OH groups and another aromatic ring with two non-adjacent phenolic OH groups.

Drawing a universal correlation between chemical structures and the obtained thicknesses in binary combinations seems to be difficult. Some binary combinations yielded a significantly higher coating thickness compared to that of the individual compounds such as combinations of **P21** and **P10** yielded a coating of 96 ± 11 nm compared to 63 ± 5 nm and 7 ± 3 nm obtained for individual **P21** and **P10**, respectively (Fig. 3 and Fig. S15). In some other combinations, the thickness of the coatings derived from binary combinations was less than that obtained from the individual compounds. An example includes a binary combination of **P21** and **P13** leading to 13 ± 2 nm coating, compared to the thickness from the individual compounds **P21** (63 ± 5 nm) and **P13** (31 ± 4 nm) (Fig. 3). Although the thickness was increased in one (**P21** + **P10**) and deceased in another combination (**P21** + **P13**), the UV absorbance of both combinations was increased significantly over time and was more than the UV absorbance of the individual compounds (Fig. 2).

In some binary combinations, keeping one component the same while varying the second component having different chemical functional groups led to a significant alteration of coating thicknesses. For example, the coating thickness obtained from binary combinations of **P21** varied between 13 ± 2 nm (for **P21** + **P13**) to 162 ± 10 nm (for **P21** + **P38**) (Fig. 3). Similarly, the thickness of nanocoatings derived from binary combinations of **P10** varied between 15 ± 3 nm (for **P10** + **P28**) and 98 ± 7 nm (for **P10** + **P6**) (Fig. 3).

In order to quantitively compare the synergistic or antagonistic effect in the combinations, we have compared the thickness values calculated based on an assumed additive behavior (average of two values) with the experimental values measured by ellipsometry. For examples discussed above, in combinations made with **P21** (dopamine), we observed synergistic effect for combinations of **P10** + **P21**, **P38** + **P21**, **P26** + **P21** and antagonistic effect in combination **P13** + **P21**, **P22** + **P21** (Fig. S16). A synergistic or antagonistic effect was observed for most of the studied combinations (Fig. S17). Combinations made with **P9** (pyrocatechol) as one component had the highest occurrence of synergistic effect (15 out of 20 combinations). Combinations made with **P23** (pyrogallol) or **P29** (5-hydroxydopamine hydrochloride) as one component showed the highest occurrence of antagonistic effect (10 out of 20 combinations) (Fig. S17).

In order to evaluate whether systematic statistical data analysis by means of machine learning (ML) reveals underlying patterns, e.g. correlations between the chemical structure of the precursor materials and final layer thickness, we trained simple ML models such as random forests based on various representations of the chemical structure of the precursors on the 140 data points shown in Fig. 3. We mainly focused our analysis on the question, which features or descriptors are most relevant to explain the experimentally observed layer thickness. For the ML tests using chemical fingerprints as features, the number of OH groups was by far the most important feature and accounts for about 30% of the variation of the thickness. Further relevant chemical groups are 3C2OH (exactly two OH groups in a ring with one carbon atom distance, e.g. 1,3-dihydroxybenzene) and COOH groups (carboxylic acid, e.g. 3,4-Dihydroxybenzoic Acid). Although the machine learning models systematically identified certain chemical features to be relevant for determining the coating thickness of a binary mixture, no single feature correlates strongly with the final coating thickness (see Fig. S18 in the SI). Only a combination of the features, which is learned by the machine learning model can predict a weak trend, but more data would be needed to improve the predictions and generate more precise design rules (for further details on ML see Supplementary Information).

UV–vis spectra and ellipsometry (Fig. 2, Fig. 3, Figs. S2–S13) demonstrate unexpected synergistic behavior in the oxidation and deposition of mixtures of phenolic compounds and catecholamines. Such non-additive behavior together with the vast diversity of available phenolic compounds highlights the importance of high-throughput screening approaches to discover the true potential of these materials.

In order to facilitate the investigation of as many as possible combinations of phenolic compounds and catecholamines, we developed a miniaturized high-throughput strategy to explore the properties of coatings derived from single, binary, and tertiary combinations. Thus, 2,532 solutions containing single, binary, and tertiary combinations of phenolic compounds were printed into predefined spots on a non-treated rectangular 4-well cell culture plate.

**Scheme 1:**
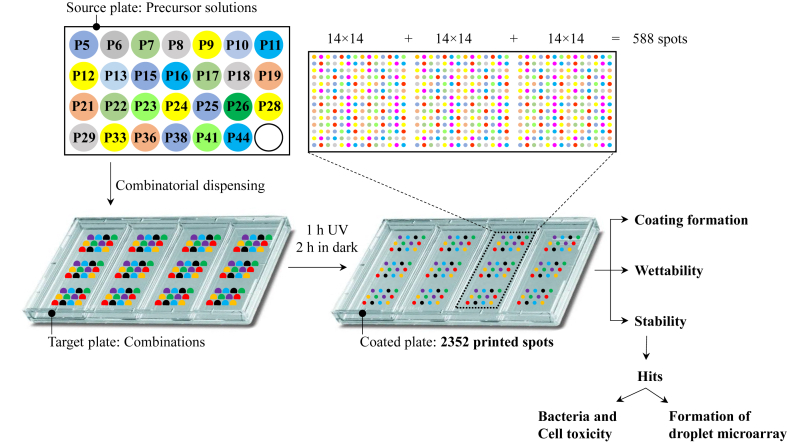
Schematic representation of the miniaturized high-throughput strategy to form nanocoatings from single, binary, and ternary combinations of 27 different phenolic compounds and catecholamines (fed into the source plate) on a standard 4-well plate. A non-contact liquid dispenser was used to print combinatorial precursor solutions to form 2,352 droplets, followed by 1 h UV irradiation (365 nm, 5 mW/cm^2^) and 2 h incubation in dark to induce polymerization and to form coating. The coating formation ability and stability of the nanocoatings were investigated. Stable coatings were used to create arrays of hydrophilic spots on hydrophobic surface of a 4-well plate, which were then evaluated for their influence on HeLa CCL2 cells and *P. aeruginosa* (strain PA30) bacteria.

The screening of nanocoatings in a high-throughput manner is shown schematically in Scheme 1 and with more details in Scheme S1. Briefly, the precursor solutions of 27 phenolic compounds (1 mg/mL, 10 mM Tris buffer, pH 8.5) were loaded in the source plate of the non-contact liquid dispenser device, single, binary (1:1) and ternary (1:1:1) combinations (volume ratios, 120 nL of single precursor solutions; each component at 1 mg/mL, 10 mM Tris buffer, pH 8.5) were printed on the surface to form an array of 2,352 droplets (588 combinations × 4 repetitions). The droplet array was placed in a humid environment, irradiated with UV light for 1 h (365 nm, 5 mW/cm^2^), followed by 2 h incubation in the dark. Combinations used to coat each spot are shown in Scheme S2.

**Fig. 4:**
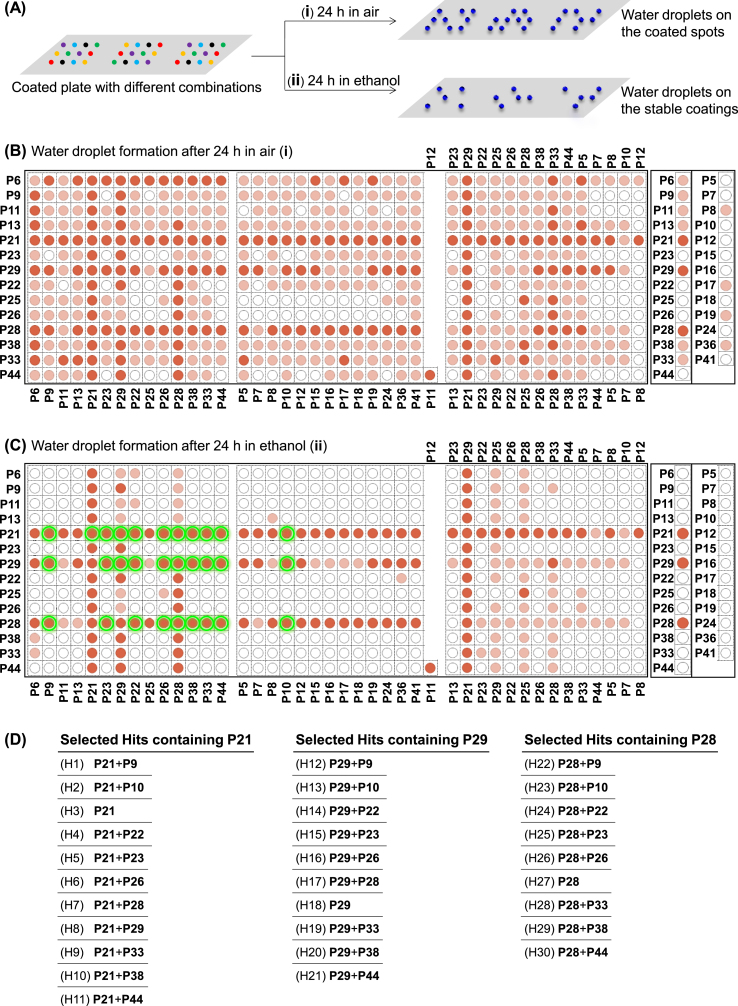
(A) Formation of water droplets was compared for the coated substrate with (i) and without (ii) immersion in ethanol for 24 in order to identify stable nanocoatings (stable hits). The heatmap of the water droplets formed on the coated substrate, without (B) and with (C) incubation in ethanol for 24 h. The intersection of each column and row is a combination made from compounds shown on the edge of the corresponding column(s) and row. Spots forming droplets with a diameter (measured and averaged from four experiments) of more than 0.55 mm are color coded in dark red, between 0.15 mm and 0.55 mm in light red, and below 0.15 are not colored. Nanocoatings that led to the formation of water droplets after ethanol treatment were identified as stable hits. Spots treated with single precursor solutions are shown separately in the right column of (B) and (C). Corresponding compounds present in each combination are also shown in a tabular format in Scheme S2. (D) Stable hits were selected for tuning surface wettability and investigation of the biological properties. The corresponding coated spots with these 30 combinations are highlighted in green color in (C).

In order to assess the formation of coatings and their stabilities in a high-throughput way, we used the ability of polyphenolic coatings to change local wettability [6,8], thereby achieving the effect of discontinuous dewetting. Discontinuous dewetting is the separation of a large liquid droplet into smaller droplets due to the large difference in spatially patterned wettability [36,37]. This method is perfectly suitable for ultra-high-throughput screening of surface functionalization, surface wettabilities, and to test the stability of surface coatings. Thus, following the coating process described above, around 5 mL drop of water was placed on the hydrophobic polystyrene (PS) substrate (advancing, receding, and static water contact angles of PS 92°, 76°, 88°, respectively) that was patterned with polyphenolic nanocoatings. By tilting the substrate, the droplet rolled off the surface, leading to the spontaneous formation of an array of smaller droplets in hydrophilic spots [36,37] (Fig. 4A, Videos S1 and S2). Bright-field microscopy images of droplet microarrays were taken and used to identified and categorized droplets based on their size. The size of the droplets was determined by ImageJ software (Fig. S19, Fig. 4B(i)). Water droplets were formed on 465 out of 588 spots treated with phenolic compounds (Fig. 4B(i), Fig. S19). To determine the stability of the nanocoatings the coated substrates were immersed in ethanol for 24 h followed by the formation of water droplets using discontinuous dewetting (Fig. 4, Fig. S21). After immersion in ethanol for 24 h water droplets were formed only on 122 spots (shown in dark red in Fig. 4C(ii)).

Supplementary video related to this article can be found at https://doi.org/10.1016/j.mtbio.2021.100108

The following is/are the supplementary data related to this article:

Video S1 Video S1

Video S2 Video S2

Spots coated with single precursor solutions are shown in a separate column on the right of Fig. 4B and C. Only three compounds **P21**, **P28**, **P29** out of the 27 compounds were able to form stable nanocoatings derived from single precursors (right columns on the right of Fig. 4C). However, most of the combinations containing **P21**, **P28**, or **P29** (row 5, 7, and 11, respectively) formed stable nanocoatings. In these three compounds, an amino group is attached to the phenolic ring (having two or three phenolic OH groups) via a –CH_2_-CH_2_– bridge. This structure has been previously considered advantageous in the polymerization and deposition of catecholamines [20].

Interestingly, we observed several synergistic effects among the stable hits. For example, **P10** could not form a stable coating when used as a sole component. However, the combination of **P10** + **P21** resulted in a stable nanocoating with a thickness of 96 ± 11 nm - more than the thickness of the individual compounds (7 ± 3 nm, 63 ± 5 nm for **P10** and **P21**, respectively, Fig. 3). Similar synergy behavior was observed for the binary precursor solution of **P38** + **P21** and a combination of **P26** + **P21**. However, some combinations such as **P10** + **P9** with new peaks observed in the UV–vis spectra (Fig. 3) did not yield stable coatings. On the other hand, some of the binary combinations formed stable nanocoatings with lower thickness compared to that formed by their single components: such as the binary combinations of **P13** + **P21** and **P22** + **P21** (Fig. 3). Thus, this method can be used to quickly identify stable coatings leading to better wettability required for the effect of discontinuous dewetting. Stable coatings leading to hydrophobic coatings, however, cannot be detected by this method.

Within the single combinations, the top five highest thickness values were obtained for **P38** (106 ± 9 nm), **P33** (92 ± 7 nm), **P21** (63 ± 5 nm), **P29** (58 ± 5 nm), **P22** (55 ± 7 nm). However, only three single combinations formed stable nanocoatings after 24 h immersion in ethanol, including **P21**, **P29**, and **P28**. Interestingly, **P28** formed stable nanocoating, although its thickness (19 ± 2 nm) was not among the largest for single combinations.

Among all studied combinations, the top five highest thickness values were obtained for binary combinations of **P21** + **P38** (162 ± 10 nm), **P9** + **P26** (126 ± 8 nm), **P21** + **P5** (115 ± 13 nm), and **P6** + **P38** (113 ± 12 nm), and **P38** (106 ± 9 nm), from which only the combination **P21** + **P38** was stable after 24 h immersion in ethanol. The five stable nanocoatings with the highest thickness values were **P21** + **P38** (162 ± 10 nm), **P21** + **P10** (96 ± 11 nm), **P21** + **P26** (87 ± 13 nm), **P29** + **P38** (82 ± 4 nm), **P21** + **P33** (76 ± 12 nm), and the top five stable nanocoatings with the lowest thickness values were **P21** + **P22** (8 ± 2 nm), **P28** + **P44** (9 ± 2 nm), **P28** + **P10** (15 ± 3 nm), **P28** (19 ± 2 nm), **P28** + **P26** (21 ± 3 nm).

Next, in order to investigate the wettability and biological properties of the stable nanocoatings, 30 hits made of combinations of **P21, P28**, and **P29** were selected for further characterization as indicated in Fig. 4D. Corresponding coated spots with these combinations are highlighted in green in Fig. 4C(ii). The thickness of the nanocoatings derived from hit combinations was measured before and after 24 h immersion in ethanol. No significant change in the thickness values of the stable hits was observed after ethanol treatment.

**Fig. 5:**
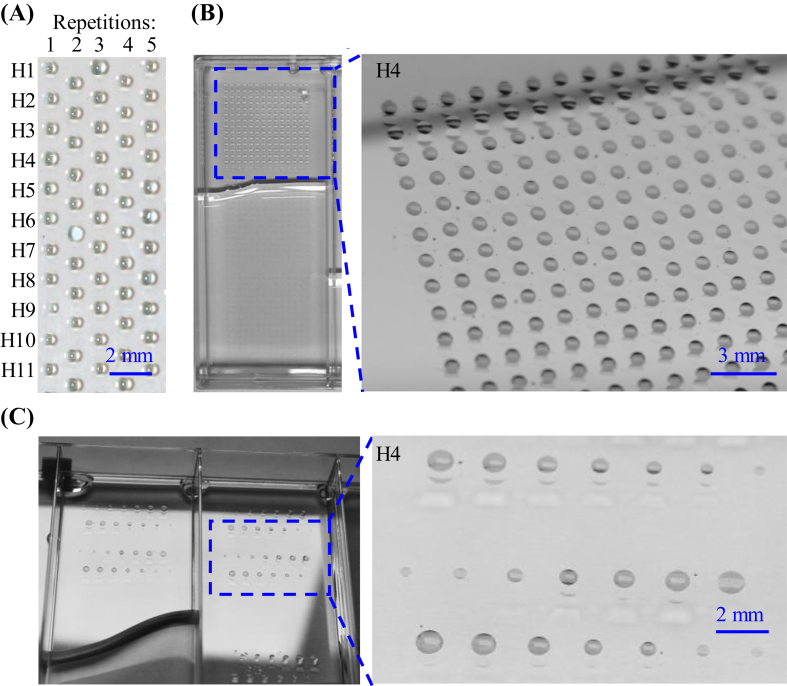
Spontaneous formation of a uniform water droplet microarray (DMA) on the polystyrene surface patterned using H1–H11 polyphenolic mixtures (see Fig. 4), (A) or on the surface coated with arrays of the hit H4 (B) through the effect of discontinuous dewetting. Spot size gradients of DMA were formed spontaneously on the surface modified with different volumes of the printed precursor of H4 (C). In all the experiments the coated substrate was immersed in ethanol for 24 h and then dipped in water to form DMA through the effect of discontinuous dewetting.

Stable hits H1–H11 identified after 24 h ethanol treatment (Fig. 4D) were used to demonstrate surface functionalization and tuning of surface wettability of polystyrene (PS) surface directly inside wells of a Petri-dish or microtiter plate. 150 nL of H1–H11 were printed on the PS surface using a non-contact liquid dispenser in predefined spots followed by 1 h UV irradiation and 2 h incubation in dark. The substrate was washed, dried, and immersed in ethanol for 24 h. Then, by immersion of the coated substrate in water and tilting it, a uniform microarray of water droplets (circles with a diameter of 750 μm) was spontaneously formed on the coated spots through the effect of discontinuous dewetting (5 columns in Fig. 5A). In another experiment, we used H4 (combination of **P21** + **P22**) to coat 588 predefined spots located in one well of a 4-well plate by printing 150 nL droplets. 588 water droplets were formed in each well via the effect of discontinuous dewetting (Fig. 5B).

To demonstrate the versatility of this approach for surface modification, a spot size gradient of water droplets was formed using mixture H4 and H29. To this end, the printed volume of the precursor solution was varied from 30 nL to 210 nL on several spots on the PS surface, followed by the same procedure. A spot size gradient of water droplets with diameters from 150 μm to 1100 μm was spontaneously formed on the surface following the dipping of the coated substrate in water (Fig. 5C for H4, Supplementary Video S3 for H29).

Supplementary video related to this article can be found at https://doi.org/10.1016/j.mtbio.2021.100108

The following is/are the supplementary data related to this article:

Video S3 Video S3

Having demonstrated the formation of stable nanocoatings derived from phenolic compounds and catecholamines, we have investigated the biological activity of the hit nanocoatings (H1–H30) by determining the metabolically active bacteria of *P. aeruginosa* strain PA30 strain, cell viability, and toxicity for human cervical adenocarcinoma HeLa cell line (HeLa CCL2) (Fig. S21). HeLa CCL2 was selected to test the biocompatibility and applicability of nanocoatings since it is a widely used eukaryotic model cell line in biomedical applications [38]. *P. aeruginosa* is one of the six bacterial pathogens (known as ESKAPE), which can grow in a wide variety of niches [26], exhibit antimicrobial resistance, and cause severe threat to human health [39]. Therefore, *P. aeruginosa* was selected to investigate the bacterial response to the nanocoatings due to its high importance in health care and biomedical applications.

To determine the activity of the coatings toward bacteria, wells of a 96 well plate were coated with the hits as describe above (H1–H30, three repetitions), followed by sterilization with ethanol. Then 200 μL of the bacterial solution of *P. aeruginosa* PA30 GFP was incubated for 24 h at 37 °C into each well of the already coated 96-well plate. Solutions of CTC (2,3-ditolyl tetrazolium chloride, 4 × 10^−3^ M, 3 h at 37 °C) and DAPI (4’,6-diamidino-2-phenylindole, 1 μg/mL, 10 min at RT) were used to stain the bacteria. Stained samples were images by AxioImage M2 system equipped with an Apotome. No toxicity of the nanocoatings towards the studied bacteria was observed after 24 h incubation at 37 °C (Figs. S21A and B). Survival rates of the bacteria on the nanocoatings were generally higher than that on the non-coated polystyrene (PS) substrate (Fig. S21A), except for H10, H23, H25, and H30 for which bacterial survival rates were slightly lower than on non-coated PS (95%, 94%, 89%, and 90%, respectively). Although antibacterial activity has been reported for some plant phenolic compounds [9,21,40,41], it was also shown that these compounds could both enhance or inhibit the formation and growth of bacteria depending on the experimental conditions [[40], [41], [42], [43], [44], [45]].

To determine the toxicity of the nanocoatings to mammalian cells, 100 μL Hela cell suspension was added into each well of the 96-well plate, which was already coated with the hit combinations (H1–H30) and incubated for 24 h at 37 °C, followed by incubation for 15 min in staining solutions of Hoechst 33342 (1 μg/mL), Calcein AM (0.5 μg/mL), and PI (0.5 μg/mL). Four images of each well were taken using Olympus IX81 inverted motorized microscope and the number of Hoechst 33342- and PI-positive cells were counted using ImageJ to calculate the cell viability: cell viability (%) = (1-PI positive cell numbers/Hoechst 33342 positive cell numbers) × 100. The results of cell viability experiments indicated that nanocoatings derived from hit combinations (H1–H30) were nontoxic to mammalian cells after 24 h incubation at 37 °C and in general, slightly enhanced the cell attachment to the surface (Fig. S21C, S22). The nanocoatings demonstrated excellent cell viabilities, which were comparable to non-coated PS surface, indicating good biocompatibility during the culture period. The cell viabilities on the treated surfaces with the hit nanocoatings were all greater than 80% (Fig. S21C), which demonstrated a non-toxic effect of nanocoatings. As shown in Fig. S22, the calcein AM-stained cells displayed the typical morphology of HeLa cells on both non-coated PS surface and nanocoatings, which was soft-irregular shaped, hyper motile, with a thin rim of cytoplasmic and projected-filamentous [46], indicating the biocompatibility and good cell adhesion property of nanocoatings.

## 4 Conclusion

In conclusion, we investigated the oxidation and deposition behavior of a 45-membered library of phenolic compounds and catecholamines under basic conditions and UV irradiation. 9 out of 45 compounds with relatively high molecular weights were not soluble at the same conditions and were not investigated further. UV–vis spectroscopy of the single-component solutions (measured for 36 soluble compounds) and ellipsometry measurements of the nanocoatings derived from them (measured for 27 single component precursor solution) indicated that oxidation and deposition behavior strongly depends on the chemical structure of phenolic compounds, number and distribution of phenolic OH groups, and presence of additional functional groups such as amines. These results also indicated that the oxidation and deposition process can be accelerated by UV irradiation.

Different UV–vis spectra and thickness values were observed for the combinations indicating potentially distinct oxidation and/or deposition mechanism of the binary mixtures. In some combinations, a synergistic (or antagonistic) effect was observed in which the thickness values of nanocoating derived from these combinations were higher (or lower) than thickness values of the single component precursors or the thickness value calculated assuming an additive behavior.

Furthermore, in order to facilitate the investigation of as many as possible combinations of phenolic compounds and catecholamines, we developed a miniaturized high-throughput strategy that helped us to rapidly explore coating properties of 2,532 spots functionalized by single, binary, and tertiary combinations of polyphenols in a single run. The coatings’ stability and formation were analyzed in a single step by the formation of water droplets using the effect of discontinuous dewetting. In each well of a 4-well polystyrene plate, water droplets were formed on 465 out of 588 spots treated with the precursor solutions from which 122 combinations were stable even after 24 h immersion in ethanol. Combinations that contained one component with an amino group attached to its phenolic ring formed the most stable hydrophilic nanocoatings. Interestingly, stable nanocoatings could be obtained from some combinations where single components failed to form stable coatings. The identified stable nanocoatings were used to create arrays of hydrophilic spots directly inside polystyrene multiwell plates for the formation of droplet microarrays, which can find applications for high-throughput biological screenings. Finally, we did not observe any strong toxicity of the selected nanocoatings in bacterial or eukaryotic cell culture experiments after 24 h incubation at 37 °C.

## Author contributions

Farid Behboodi-Sadabad conceived the idea and developed the miniaturized high-throughput process to form nanocoatings using precursors based on phenols and catecholamines and wrote the manuscript. Farid Behboodi-Sadabad performed the UV–vis spectroscopy and ellipsometry analysis. Shuai Li performed the stability experiments and prepared the spot size gradients of droplet microarrays. Lei Wenxi performed the antimicrobial experiments. Yanxi Liu performed cell toxicity experiments. Timo Sommer and Pascal Friederich designed and performed the machine learning tests and models. Caroline Sobek helped in performing UV–vis spectroscopy experiments. All coauthors discussed the paper and revised the manuscript. Farid Behboodi-Sadabad and Pavel Levkin were involved in designing the experiments. Farid Behboodi-Sadabad, Phillip Messersmith, and Pavel Levkin supervised the work.

## Declaration of competing interest

The authors declare that they have no financial interests or personal relationships that could have appeared to influence the work reported in this paper.
