# Supplementary material for: High-throughput screening of multifunctional nanocoatings based on combinations of polyphenols and catecholamines
Source: Mater Today Bio. 2021 Mar 10;10:100108. doi: 10.1016/j.mtbio.2021.100108 (PMC8063910; doi:10.1016/j.mtbio.2021.100108)
Supplement: Multimedia component 1 [file mmc1.pdf]

Supplementary Information

High-throughput Screening of Multifunctional Nanocoatings Based on Combinations of Polyphenols and Catecholamines

Farid Behboodi-Sadabad,[1]* Shuai Li,[1] Wenxi Lei,[1] Yanxi Liu,[1] Timo Sommer,[2] Pascal Friederich,[2,3] Caroline Sobek,[4] Phillip B. Messersmith,[4,5] Pavel A. Levkin[1]*

[1]Institute of Biological and Chemical Systems – Functional Molecular Systems (IBCS-FMS), Karlsruhe Institute of Technology (KIT), 76344 Eggenstein-Leopoldshafen, Germany

[2]Institute of Theoretical Informatics, Karlsruhe Institute of Technology (KIT), Am Fasanengarten 5, 76131 Karlsruhe, Germany

[3]Institute of Nanotechnology, Karlsruhe Institute of Technology (KIT), Hermann-von-Helmholtz-Platz 1, 76344 Eggenstein-Leopoldshafen,Germany

[4]Departments of Bioengineering and Materials Science and Engineering, University of California Berkeley, CA 94720-1760, USA

[5]Materials Sciences Division, Lawrence Berkeley National Laboratory, Berkeley, CA 94720, USA

E-mail address: pavel.levkin@kit.edu, farid.sadabad@kit.edu

1. Supplementary information

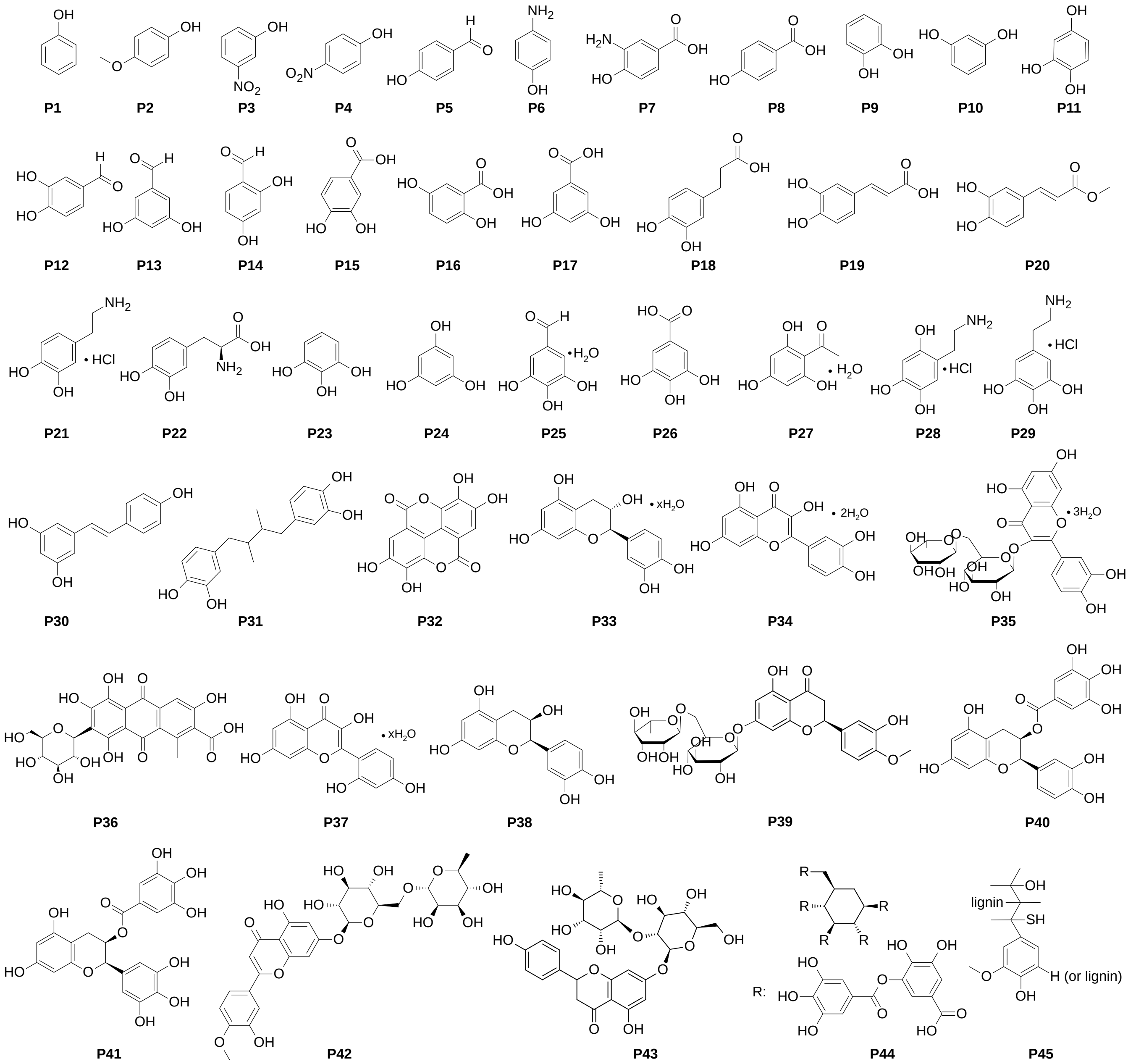

Figure S 1 Chemical structure of the compounds (phenols and catecholamines) investigated in this study.

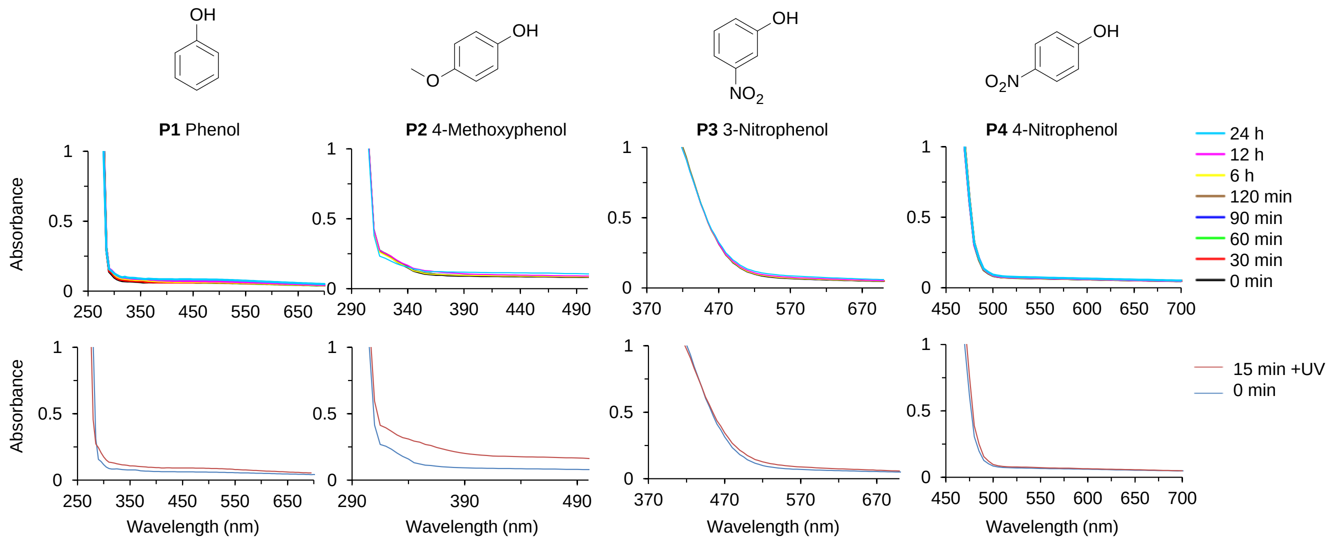

Figure S 2. UV-vis spectra of the single-component solution of the precursor compounds at pH 8.5 stored in dark environment for 24 h (top row) or after UV irradiation for 15 min (320 – 450 nm, 400 Watt, metal halide bulb, Dymax, Model 2000 Flood). Note: Sharp spikes observed around 315 nm is an artifact caused by switching the light source of the instrument at this wavelength (not originated from chemical compounds).

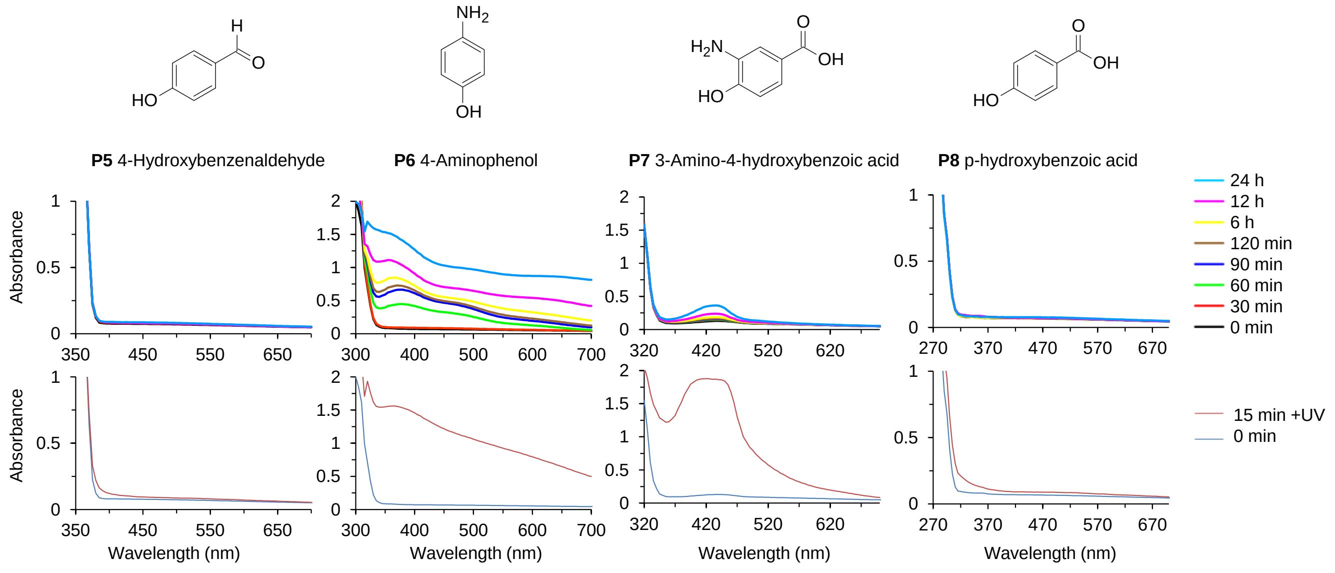

Figure S 3. UV-vis spectra of the single-component solution of the precursor compounds at pH 8.5 stored in dark environment for 24 h (top row) or after UV irradiation for 15 min (320 – 450 nm, 400 Watt, metal halide bulb, Dymax, Model 2000 Flood). Note: Sharp spikes observed around 315 nm is an artifact caused by switching the light source of the instrument at this wavelength (not originated from chemical compounds).

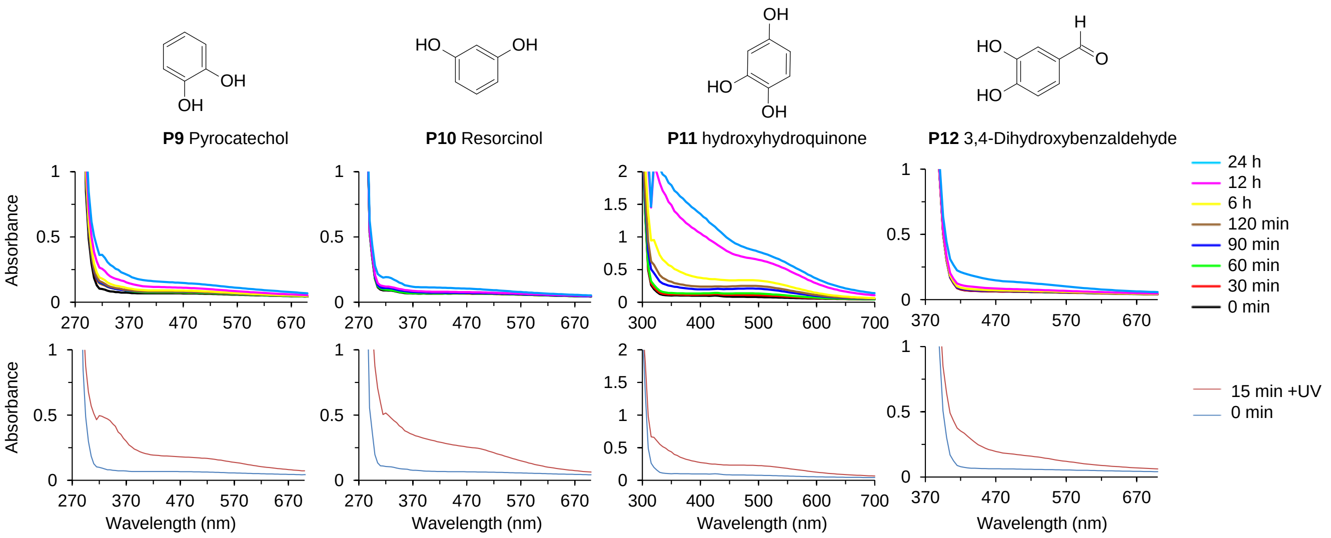

Figure S 4. UV-vis spectra of the single-component solution of the precursor compounds at pH 8.5 stored in dark environment for 24 h (top row) or after UV irradiation for 15 min (320 – 450 nm, 400 Watt, metal halide bulb, Dymax, Model 2000 Flood). Note: Sharp spikes observed around 315 nm is an artifact caused by switching the light source of the instrument at this wavelength (not originated from chemical compounds).

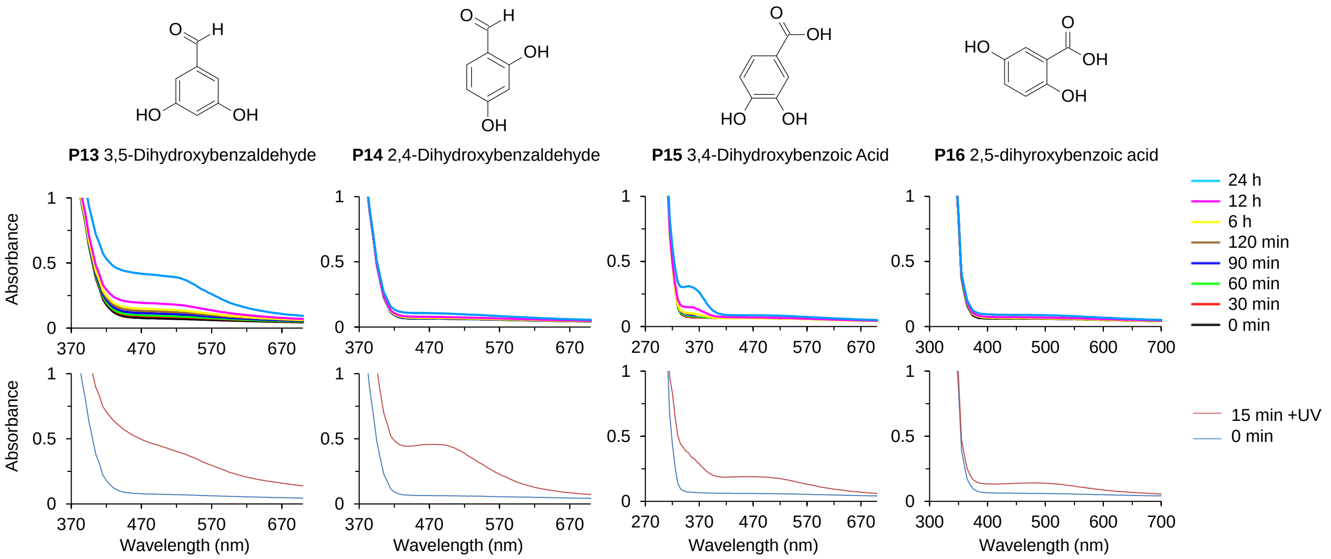

Figure S 5. UV-vis spectra of the single-component solution of the precursor compounds at pH 8.5 stored in dark environment for 24 h (top row) or after UV irradiation for 15 min (320 – 450 nm, 400 Watt, metal halide bulb, Dymax, Model 2000 Flood).

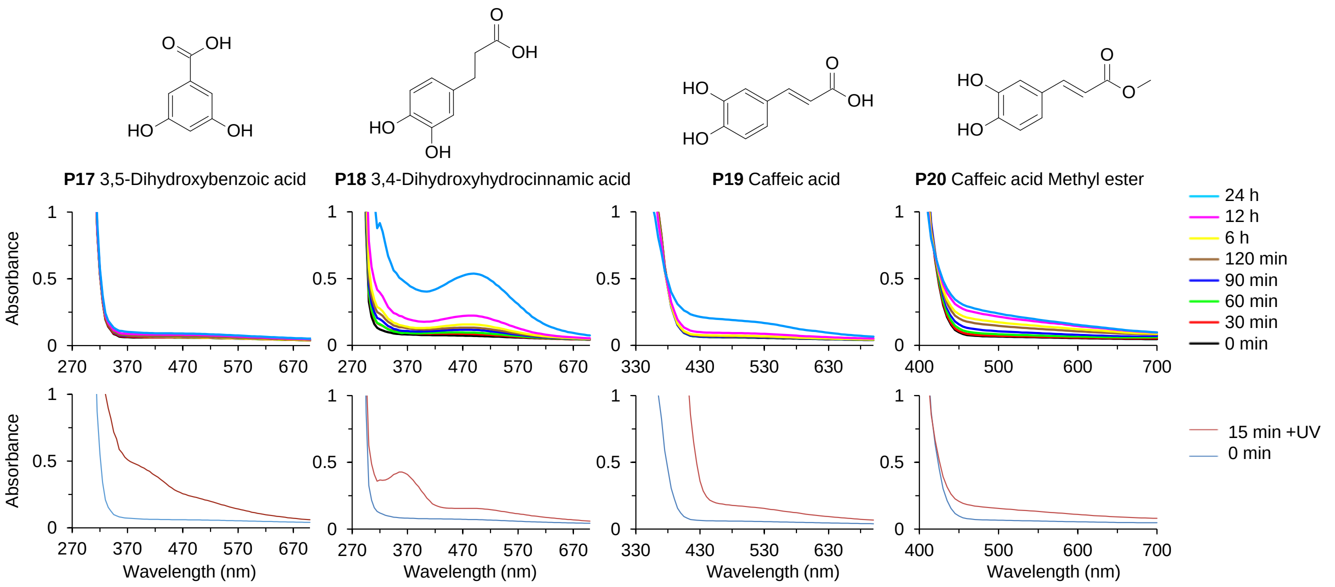

Figure S 6 UV-vis spectra of the single-component solution of the precursor compounds at pH 8.5 stored in dark environment for 24 h (top row) or after UV irradiation for 15 min (320 – 450 nm, 400 Watt, metal halide bulb, Dymax, Model 2000 Flood). Note: Sharp spikes observed around 315 nm is an artifact caused by switching the light source of the instrument at this wavelength (not originated from chemical compounds).

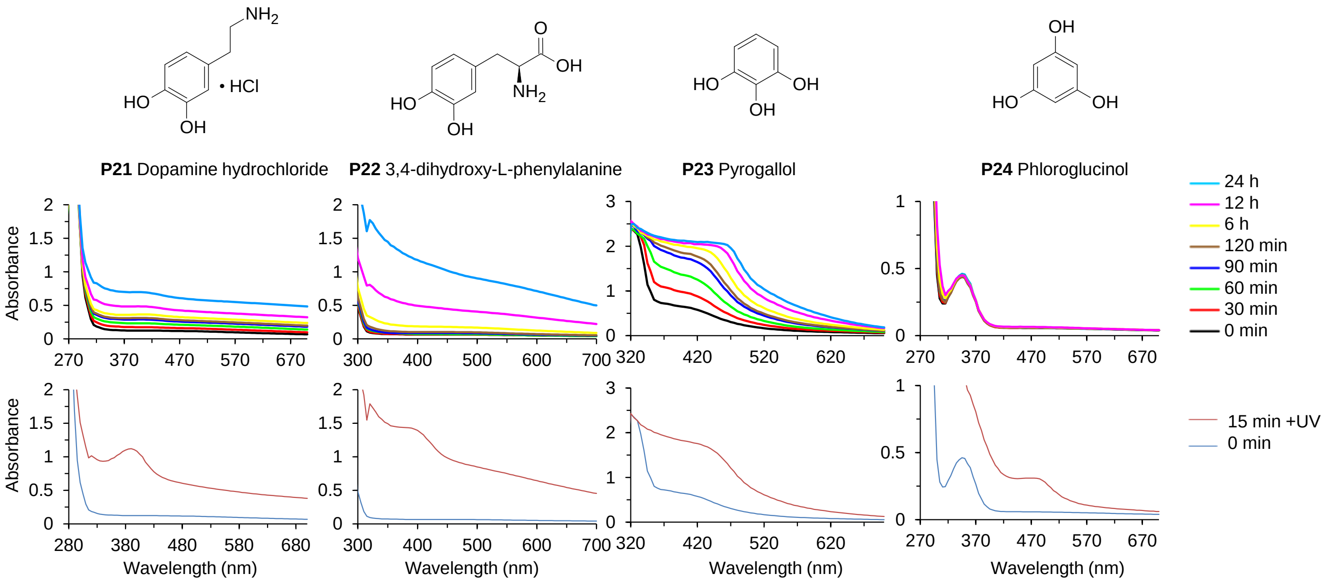

Figure S 7. UV-vis spectra of the single-component solution of the precursor compounds at pH 8.5 stored in dark environment for 24 h (top row) or after UV irradiation for 15 min (320 – 450 nm, 400 Watt, metal halide bulb, Dymax, Model 2000 Flood). Note: Sharp spikes observed around 315 nm is an artifact caused by switching the light source of the instrument at this wavelength (not originated from chemical compounds).

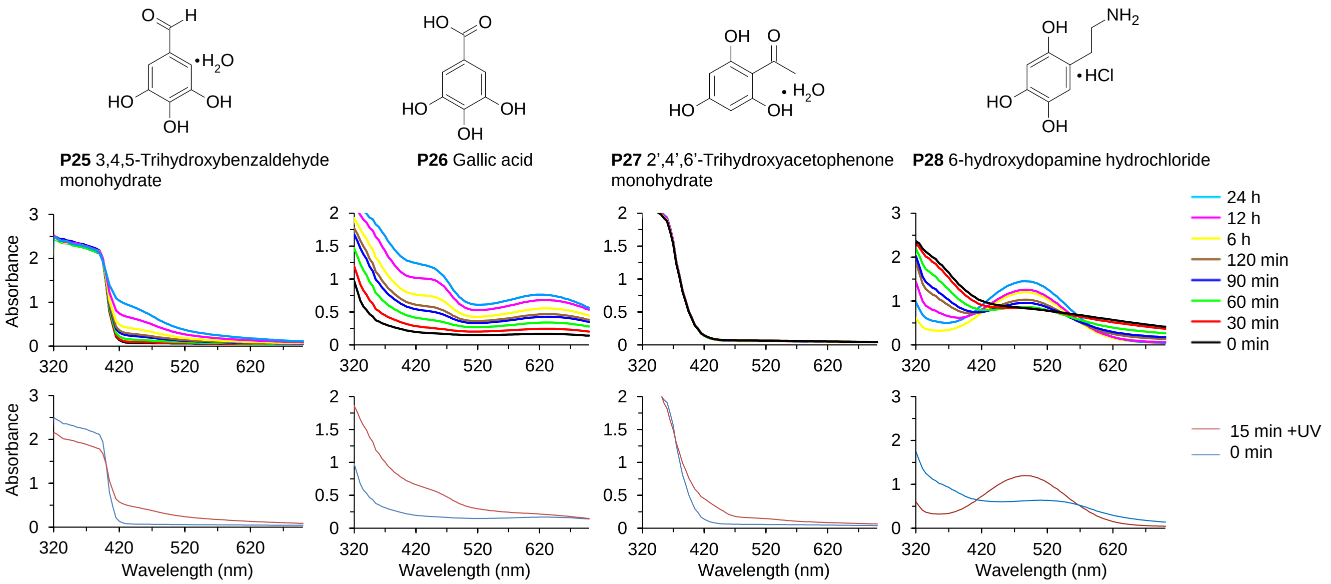

Figure S 8. UV-vis spectra of the single-component solution of the precursor compounds at pH 8.5 stored in dark environment for 24 h (top row) or after UV irradiation for 15 min (320 – 450 nm, 400 Watt, metal halide bulb, Dymax, Model 2000 Flood).

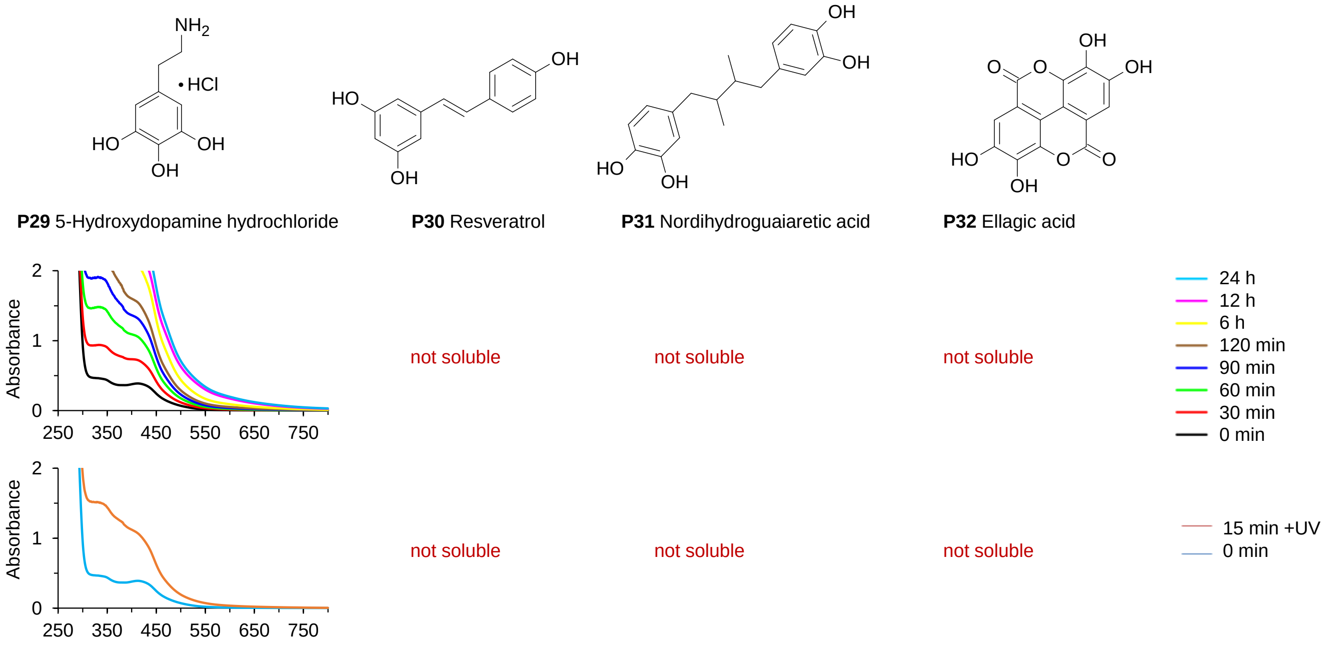

Figure S 9. UV-vis spectra of the single-component solution of the precursor compounds at pH 8.5 stored in dark environment for 24 h (top row) or after UV irradiation for 15 min (320 – 450 nm, 400 Watt, metal halide bulb, Dymax, Model 2000 Flood).

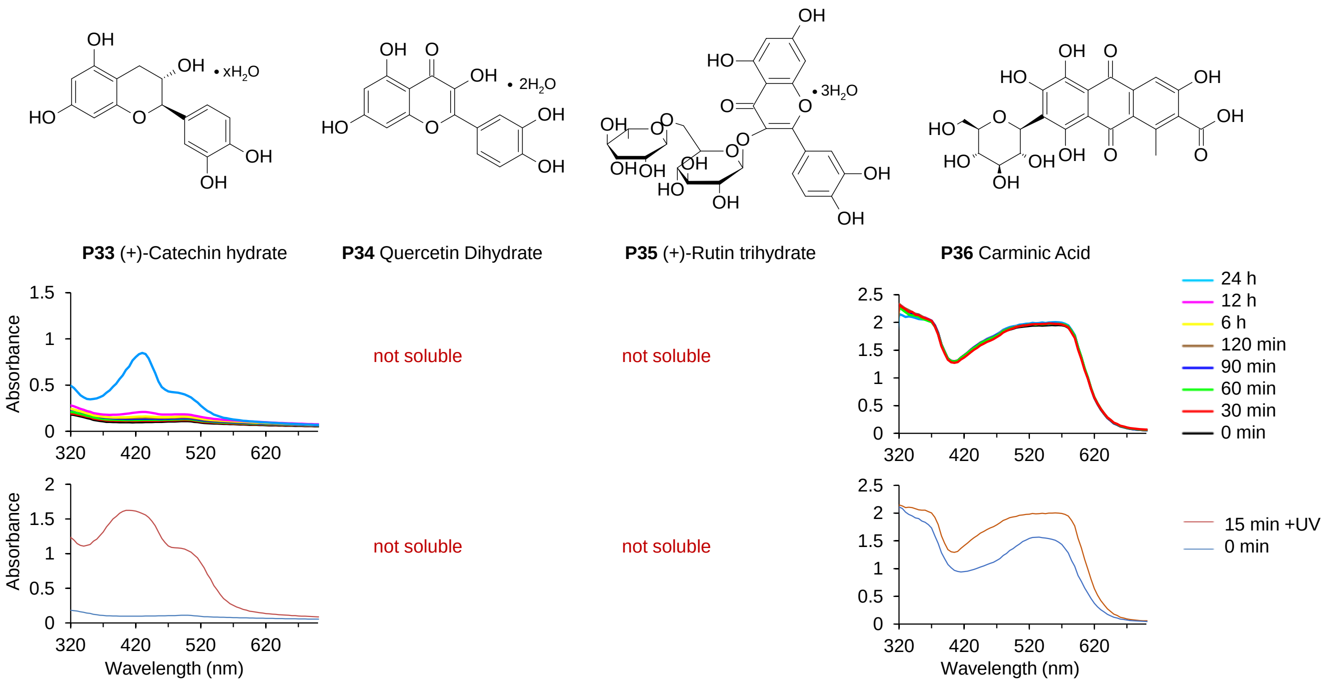

Figure S 10. UV-vis spectra of the single-component solution of the precursor compounds at pH 8.5 stored in dark environment for 24 h (top row) or after UV irradiation for 15 min (320 – 450 nm, 400 Watt, metal halide bulb, Dymax, Model 2000 Flood).

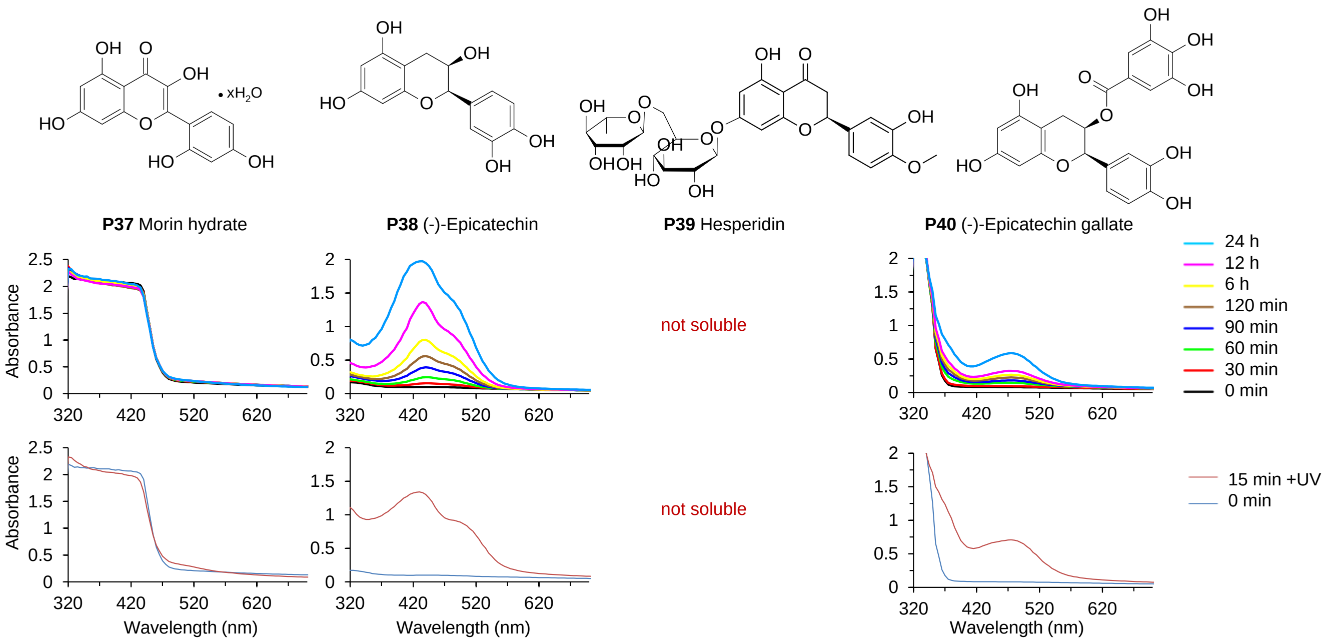

Figure S 11. UV-vis spectra of the single-component solution of the precursor compounds at pH 8.5 stored in dark environment for 24 h (top row) or after UV irradiation for 15 min (320 – 450 nm, 400 Watt, metal halide bulb, Dymax, Model 2000 Flood).

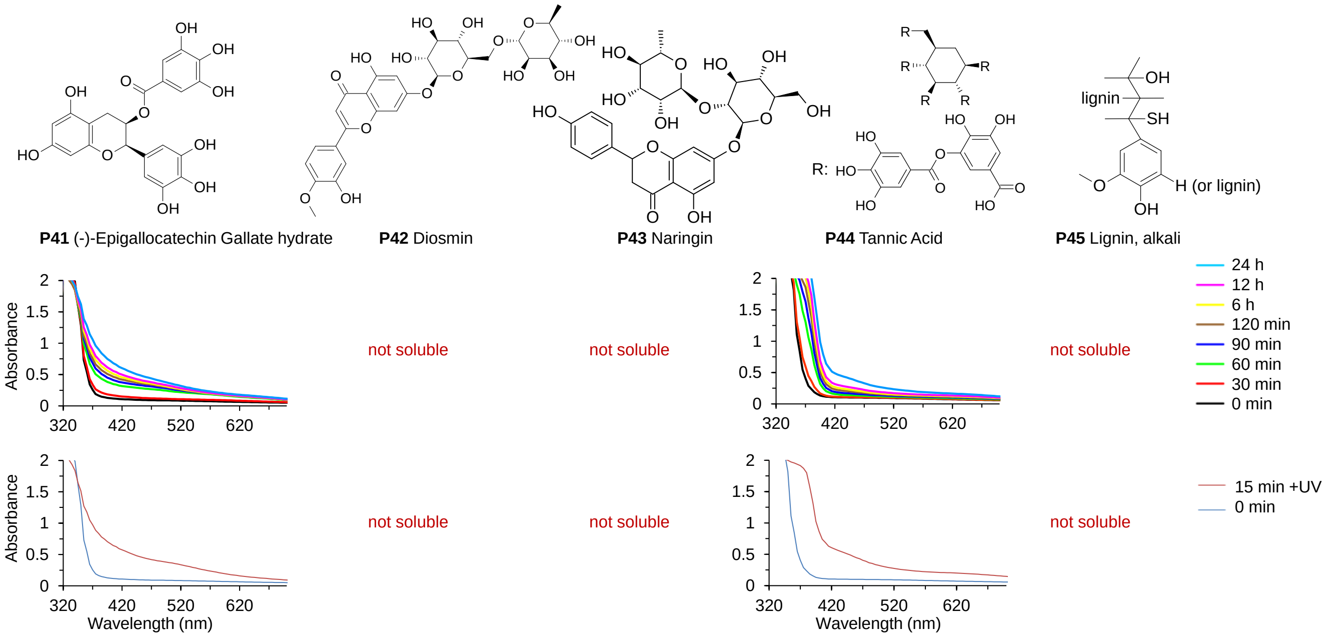

Figure S 12. UV-vis spectra of the single-component solution of the precursor compounds at pH 8.5 stored in dark environment for 24 h (top row) or after UV irradiation for 15 min (320 – 450 nm, 400 Watt, metal halide bulb, Dymax, Model 2000 Flood).

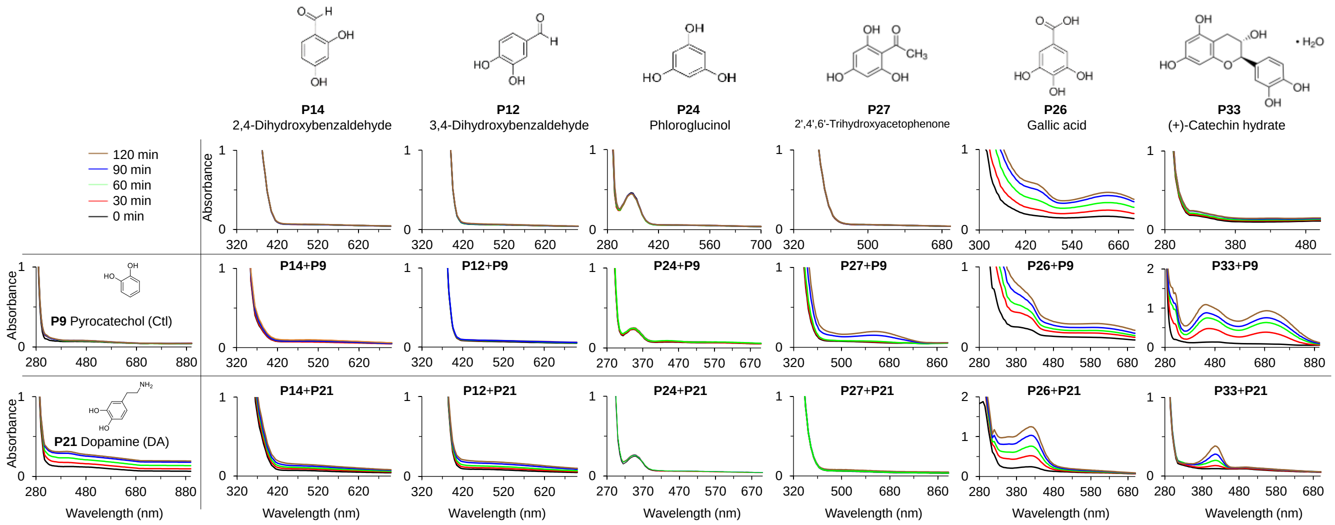

Figure S 13. Oxidation behavior of selected combinations of compounds. UV-vis spectra of the combination of **P9** (pyrocatechol) and **P21** (dopamine) with six other compounds (**P14**, **P12**, **P24**, **P27**, **P26**, **P33**) are shown. The appearance of new peaks and alteration of the intensity of the characteristic peaks of the individual compounds indicate a possible different oxidation pathway and oxidation kinetics for the combinations compared to the individual compounds.

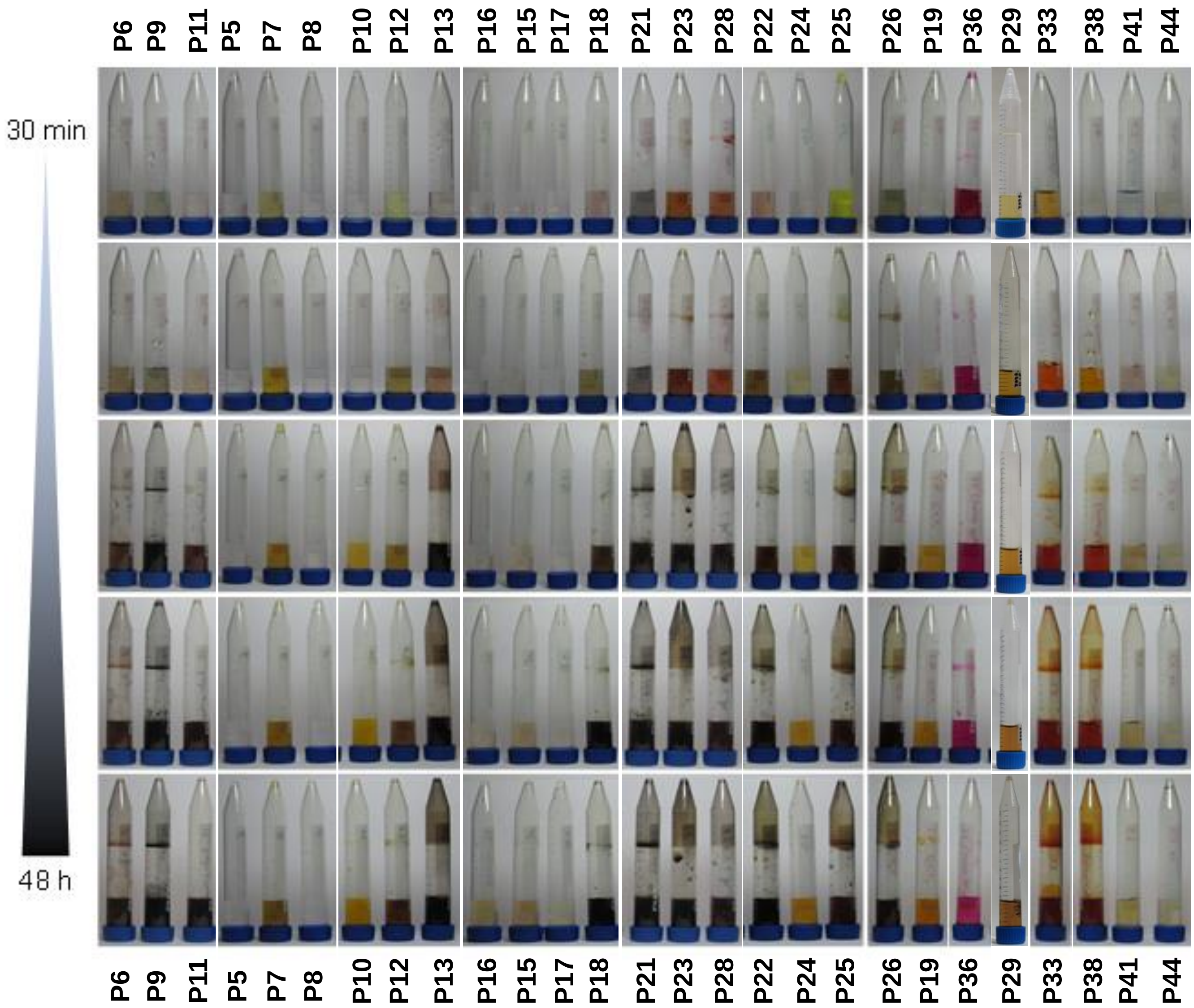

Figure S 14. Photographs of falcon tubes containing single-component solutions of precursor compounds stored in dark for 48 h at pH 8.5. The tubes were turned upside down (blue cap at the bottom) for taking photographs after 30 min, 6 h, 12 h, 24 h, and 48 h. After taking the photographs the tubes were turned again and stored in dark (with the cap on top).

No.	Chemical name	Autooxidation	Thickness (nm)
**P1**	Phenol	No	
**P2**	4-Methoxy phenol	No	
**P3**	3-Nitrophenol	No	
**P4**	4-Nitrophenol	No	
**P5**	4-Hydroxybenzaldehyde	No	0±1
**P6**	4-Aminophenol	Yes	12±3
**P7**	3-Amino-4-hydroxybenzoic acid	Yes	10±2
**P8**	*p*-Hydroxybenzoic acid	No	0±2
**P9**	Pyrocatechol (Ctl)	Yes	21±6
**P10**	Resorcinol	Yes	7±3
**P11**	Hydroxyhydroquinone (HHQ)	Yes	5±1
**P12**	3,4-Dihydroxybenzaldehyde	Yes	4±2
**P13**	3,5-Dihydroxybenzaldehyde	Yes	31±4
**P14**	2,4-Dihydroxybenzaldehyde	No	
**P15**	3,4-Dihydroxybenzoic Acid	Yes	8±1
**P16**	2,5-Dihydroxybenzoic acid	No	7±2
**P17**	3,5-Dihydroxybenzoic acid	No	4±1
**P18**	3,4-Dihydroxyhydrocinnamic acid	Yes	11±2
**P19**	Caffeic acid (CA)	Yes	8±2
**P20**	Caffeic acid methyl ester	Yes	
**P21**	Dopamine (DA)	Yes	63±5
**P22**	3,4-Dihydroxy-L-phenylalanine (DOPA)	Yes	55±7
**P23**	Pyrogallol (PG)	Yes	48±4
**P24**	Phloroglucinol	No	9±2
**P25**	3,4,5-Trihydroxybenzaldehyde monohydrate	Yes	43±5
**P26**	Gallic acid (GA)	Yes	31±4
**P27**	2-Acetylphloroglucinol	No	
**P28**	6-Hydroxydopamine hydrochloride	Yes	19±2
**P29**	5-Hydroxydopamine hydrochloride (PAE)	Yes	58±5
**P30**	Resveratrol	Not soluble	
**P31**	Nordihydroguaiaretic acid	Not soluble	
**P32**	Ellagic acid	Not soluble	
**P33**	(+)-Catechin hydrate	Yes	92±7
**P34**	Quercetin	Not soluble	
**P35**	(+)-Rutin trihydrate	Not soluble	
**P36**	Carminic acid	No	11±2
**P37**	Morin hydrate	No	
**P38**	(-)-Epicatechin	Yes	106±9
**P39**	Hesperidin	Not soluble	
**P40**	(-)-Epicatechin gallate	Yes	
**P41**	(-)-Epigallocatechin gallate hydrate	Yes	25±6
**P42**	Diosmin	Not soluble	
**P43**	Naringin	Not soluble	
**P44**	Tannic acid (TA)	Yes	10±2
**P45**	Lignin, alkali	Not soluble	

Table S 1. Summary of the oxidation behavior and the thickness values of the individual compounds. Autooxidation of the compounds in basic solution (1 mg/mL, 10 mM Tris buffer, pH 8.5) stored in dark environment, measured by UV-vis spectroscopy. A significant increase in the UV absorbance after 2 h storage in dark environment was considered as yes for autooxidation ability. The thicknesses of the nanocoatings on titanium dioxide surfaces were obtained by ellipsometry using solutions of single compounds on titanium dioxide surface. An average thickness was obtained from three independent experiments. The error bars represent ± of one standard deviation.

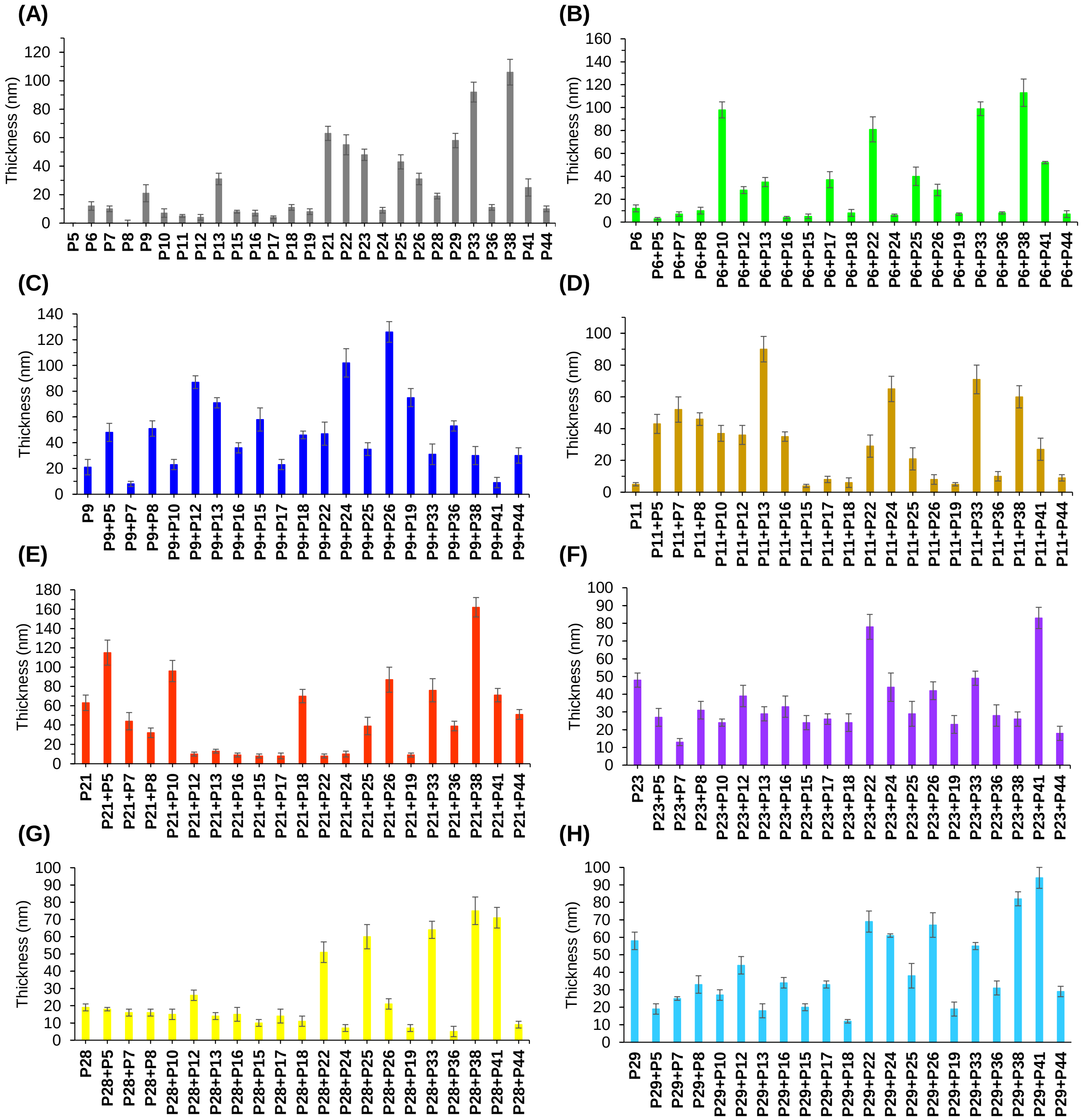

Figure S 15. Thickness (nm) of the nanocoatings derived from precursor solution of 27 selected compounds and some of their 1:1 (volume ratio of precursor solutions) binary combinations measured on titanium dioxide surface after 1 h UV irradiation and 2 h incubation in dark. The thickness values of the nanocoatings derived from the precursor solution of the single-component solutions (A) and their combinations with **P6** (B), **P9** (C), **P11** (D), **P21** (E), **P23** (F), **P28** (G), **P29** (H). An average thickness was obtained from three independent experiments. The error bars represent ± of one standard deviation.

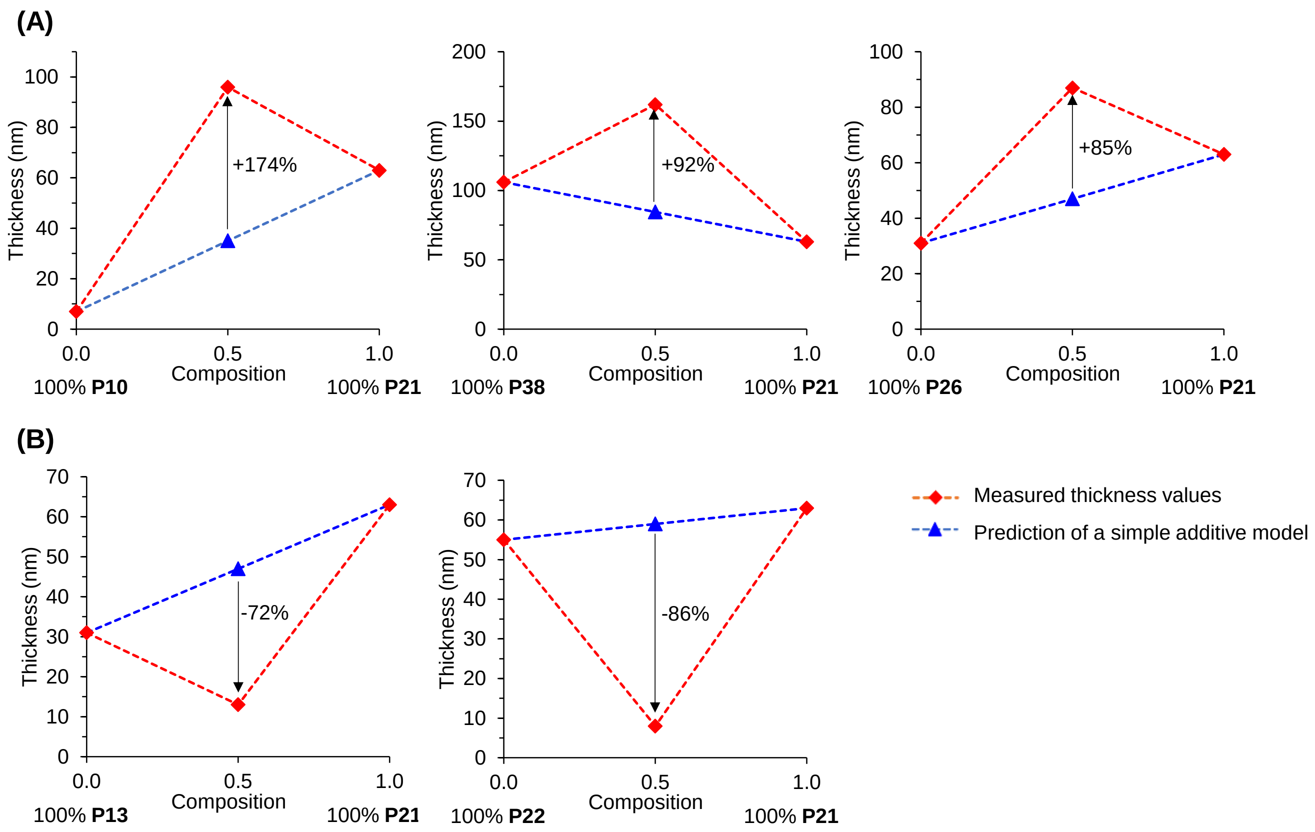

Figure S 16. Some examples of synergistic or antagonistic effect observed in thickness values of combinations. Blue triangle represents the thickness value that is expected in case of additive behavior (independent behavior) of the binary combination which is the average of two values. (Note: A constant volume of 120 nL was used to make all the combinations. Therefore, in a binary combination 60 nL of each of two components were added together). The red squares are the measured thickness values of coatings obtained from precursor solution of single and binary (1:1) combinations. In contrast to prediction from the additive model, compounds act synergistically (A) or antagonistically (B) in combinations and don't conform to the additive model. Difference in thickness values between additive model and measured data are shown in percentage on each graph.

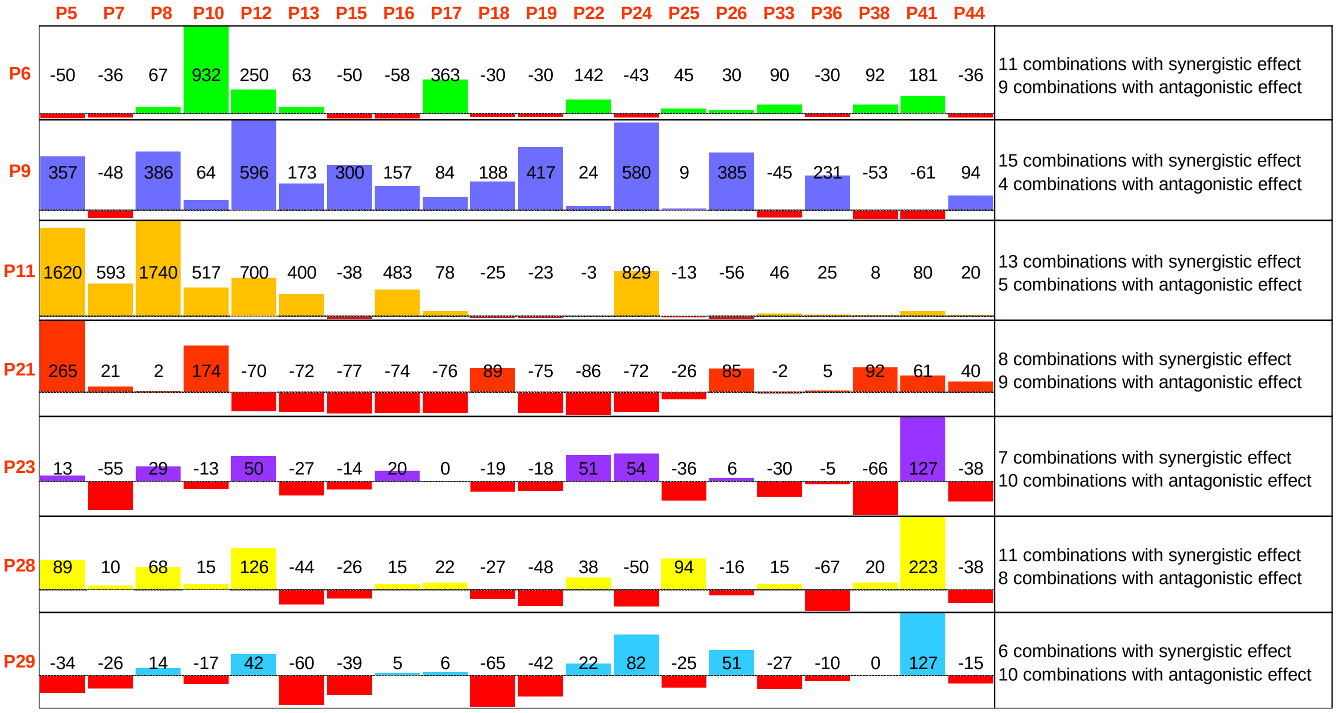

Figure S 17. Quantified representation of synergistic or antagonistic effect observed in thickness values for all studied combinations shown in Figure 3. Deviation of measured thickness by ellipsometry from the possible additive model for binary combinations is shown in percentage (%) at the intersections of the corresponding column and row. Combinations with more than 10% deviation are considered to have synergistic (> +10%) or antagonistic (< -10%) effect. The height of the columns in each row is normalized to the maximum value of deviation percentage in the same row.

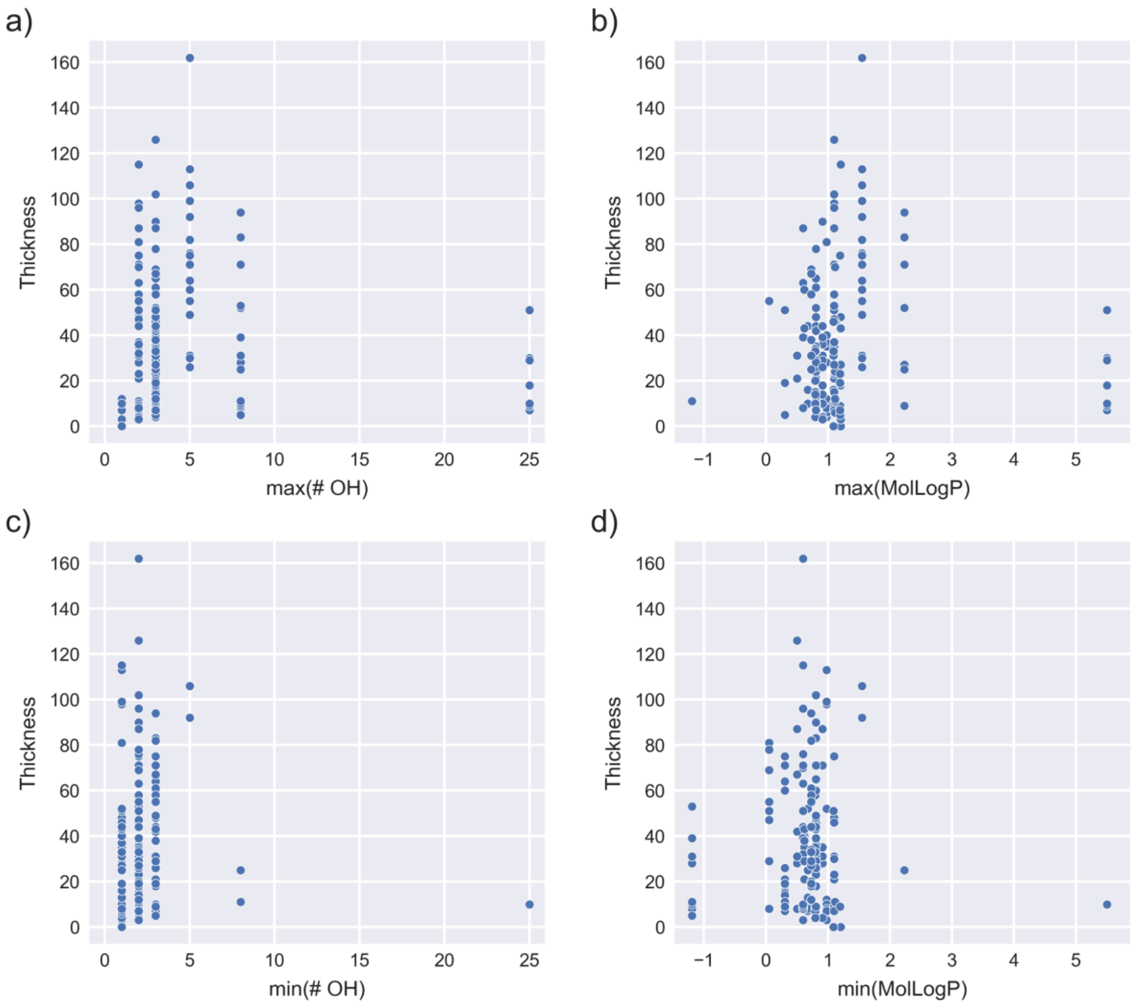

Figure S 18. Correlation plots between the thickness of binary coatings and the two molecular features that were identified as the most important ones (number of OH groups in panels (a) and (c) and logP values in panels (b) and (d)). To symmetrize the influence of the two constituents of a binary mixture, we plotted the maximal and minimal number of OH groups and logP values, respectively. As discussed in the manuscript, no single feature correlates strongly with the thickness, which is illustrated here by the absence of a clear correlation. Only the combination of features learned by the ML models can predict a weak trend between molecular structure and coating thickness.

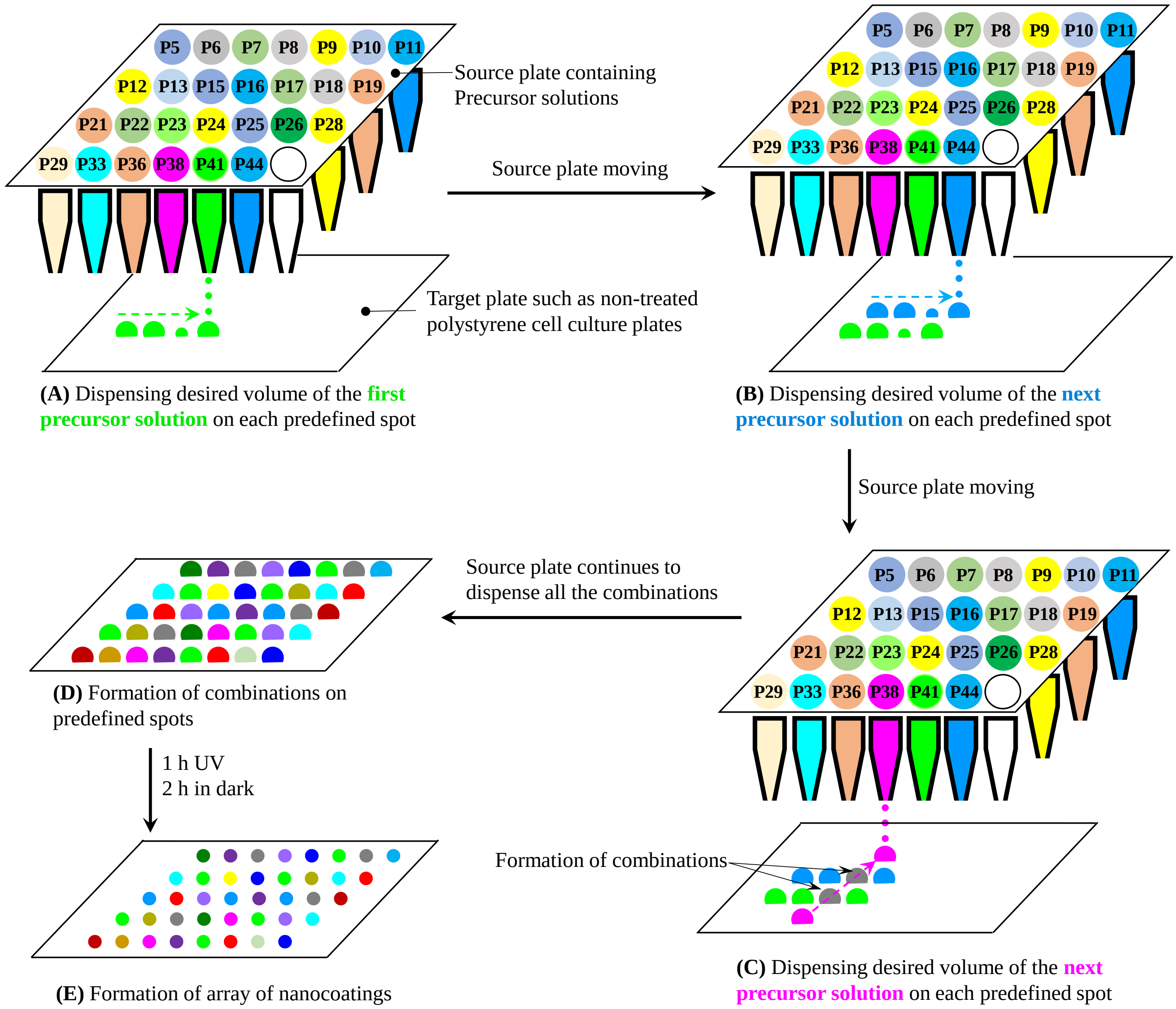

Scheme S 1. Schematic illustration of combinatorial dispensing of desired volume of the precursor solutions from the source plate onto the predefined spots on surface of the target substrate. Final dispensed volume for all combinations was kept constant at 120 nL: 120 nL for single combinations, 60 nL for each component of the binary combinations (120 nL of 1:1 volume ratio), and 40 nL for each component of the ternary combinations (120 nL of 1:1:1 volume ratio).

	1	2	3	4	5	6	7	8	9	10	11	12	13	14
1	P6 P6	P6 P9	P6 P11	P6 P13	P6 P21	P6 P23	P6 P29	P6 P22	P6 P25	P6 P26	P6 P28	P6 P38	P6 P33	P6 P44
2	P9 P6	P9 P9	P9 P11	P9 P13	P9 P21	P9 P23	P9 P29	P9 P22	P9 P25	P9 P26	P9 P28	P9 P38	P9 P33	P9 P44
3	P11 P6	P11 P9	P11 P11	P11 P13	P11 P21	P11 P23	P11 P29	P11 P22	P11 P25	P11 P26	P11 P28	P11 P38	P11 P33	P11 P44
4	P13 P6	P13 P9	P13 P11	P13 P13	P13 P21	P13 P23	P13 P29	P13 P22	P13 P25	P13 P26	P13 P28	P13 P38	P13 P33	P13 P44
5	P21 P6	P21 P9	P21 P11	P21 P13	P21 P21	P21 P23	P21 P29	P21 P22	P21 P25	P21 P26	P21 P28	P21 P38	P21 P33	P21 P44
6	P23 P6	P23 P9	P23 P11	P23 P13	P23 P21	P23 P23	P23 P29	P23 P22	P23 P25	P23 P26	P23 P28	P23 P38	P23 P33	P23 P44
7	P29 P6	P29 P9	P29 P11	P29 P13	P29 P21	P29 P23	P29 P29	P29 P22	P29 P25	P29 P26	P29 P28	P29 P38	P29 P33	P29 P44
8	P22 P6	P22 P9	P22 P11	P22 P13	P22 P21	P22 P23	P22 P29	P22 P22	P22 P25	P22 P26	P22 P28	P22 P38	P22 P33	P22 P44
9	P25 P6	P25 P9	P25 P11	P25 P13	P25 P21	P25 P23	P25 P29	P25 P22	P25 P25	P25 P26	P25 P28	P25 P38	P25 P33	P25 P44
10	P26 P6	P26 P9	P26 P11	P26 P13	P26 P21	P26 P23	P26 P29	P26 P22	P26 P25	P26 P26	P26 P28	P26 P38	P26 P33	P26 P44
11	P28 P6	P28 P9	P28 P11	P28 P13	P28 P21	P28 P23	P28 P29	P28 P22	P28 P25	P28 P26	P28 P28	P28 P38	P28 P33	P28 P44
12	P38 P6	P38 P9	P38 P11	P38 P13	P38 P21	P38 P23	P38 P29	P38 P22	P38 P25	P38 P26	P38 P28	P38 P38	P38 P33	P38 P44
13	P33 P6	P33 P9	P33 P11	P33 P13	P33 P21	P33 P23	P33 P29	P33 P22	P33 P25	P33 P26	P33 P28	P33 P38	P33 P33	P33 P44
14	P44 P6	P44 P9	P44 P11	P44 P13	P44 P21	P44 P23	P44 P29	P44 P22	P44 P25	P44 P26	P44 P28	P44 P38	P44 P33	P44 P44

	1	2	3	4	5	6	7	8	9	10	11	12	13	14
1	P6 P5	P6 P7	P6 P8	P6 P10	P6 P12	P6 P15	P6 P16	P6 P17	P6 P18	P6 P19	P6 P24	P6 P36	P6 P41	P5 P5
2	P9 P5	P9 P7	P9 P8	P9 P10	P9 P12	P9 P15	P9 P16	P9 P17	P9 P18	P9 P19	P9 P24	P9 P36	P9 P41	P7 P7
3	P11 P5	P11 P7	P11 P8	P11 P10	P11 P12	P11 P15	P11 P16	P11 P17	P11 P18	P11 P19	P11 P24	P11 P36	P11 P41	P8 P8
4	P13 P5	P13 P7	P13 P8	P13 P10	P13 P12	P13 P15	P13 P16	P13 P17	P13 P18	P13 P19	P13 P24	P13 P36	P13 P41	P10 P10
5	P21 P5	P21 P7	P21 P8	P21 P10	P21 P12	P21 P15	P21 P16	P21 P17	P21 P18	P21 P19	P21 P24	P21 P36	P21 P41	P12 P12
6	P23 P5	P23 P7	P23 P8	P23 P10	P23 P12	P23 P15	P23 P16	P23 P17	P23 P18	P23 P19	P23 P24	P23 P36	P23 P41	P15 P15
7	P29 P5	P29 P7	P29 P8	P29 P10	P29 P12	P29 P15	P29 P16	P29 P17	P29 P18	P29 P19	P29 P24	P29 P36	P29 P41	P16 P16
8	P22 P5	P22 P7	P22 P8	P22 P10	P22 P12	P22 P15	P22 P16	P22 P17	P22 P18	P22 P19	P22 P24	P22 P36	P22 P41	P17 P17
9	P25 P5	P25 P7	P25 P8	P25 P10	P25 P12	P25 P15	P25 P16	P25 P17	P25 P18	P25 P19	P25 P24	P25 P36	P25 P41	P18 P18
10	P26 P5	P26 P7	P26 P8	P26 P10	P26 P12	P26 P15	P26 P16	P26 P17	P26 P18	P26 P19	P26 P24	P26 P36	P26 P41	P19 P19
11	P28 P5	P28 P7	P28 P8	P28 P10	P28 P12	P28 P15	P28 P16	P28 P17	P28 P18	P28 P19	P28 P24	P28 P36	P28 P41	P24 P24
12	P38 P5	P38 P7	P38 P8	P38 P10	P38 P12	P38 P15	P38 P16	P38 P17	P38 P18	P38 P19	P38 P24	P38 P36	P38 P41	P36 P36
13	P33 P5	P33 P7	P33 P8	P33 P10	P33 P12	P33 P15	P33 P16	P33 P17	P33 P18	P33 P19	P33 P24	P33 P36	P33 P41	P41 P41
14	P44 P5	P44 P7	P44 P8	P44 P10	P44 P12	P44 P15	P44 P16	P44 P17	P44 P18	P44 P19	P44 P24	P44 P36	P44 P41	P44 P11 P21

	1	2	3	4	5	6	7	8	9	10	11	12	13	14
1	P6 P13 P23	P6 P21 P29	P6 P23 P22	P6 P29 P25	P6 P22 P26	P6 P25 P28	P6 P26 P38	P6 P28 P33	P6 P38 P44	P6 P33 P5	P6 P44 P7	P6 P5 P8	P6 P7 P10	P6 P8 P12
2	P9 P13 P23	P9 P21 P29	P9 P23 P22	P9 P29 P25	P9 P22 P26	P9 P25 P28	P9 P26 P38	P9 P28 P33	P9 P38 P44	P9 P33 P5	P9 P44 P7	P9 P5 P8	P9 P7 P10	P9 P8 P12
3	P11 P13 P23	P11 P21 P29	P11 P23 P22	P11 P29 P25	P11 P22 P26	P11 P25 P28	P11 P26 P38	P11 P28 P33	P11 P38 P44	P11 P33 P5	P11 P44 P7	P11 P5 P8	P11 P7 P10	P11 P8 P12
4	P13 P13 P23	P13 P21 P29	P13 P23 P22	P13 P29 P25	P13 P22 P26	P13 P25 P28	P13 P26 P38	P13 P28 P33	P13 P38 P44	P13 P33 P5	P13 P44 P7	P13 P5 P8	P13 P7 P10	P13 P8 P12
5	P21 P13 P23	P21 P21 P29	P21 P23 P22	P21 P29 P25	P21 P22 P26	P21 P25 P28	P21 P26 P38	P21 P28 P33	P21 P38 P44	P21 P33 P5	P21 P44 P7	P21 P5 P8	P21 P7 P10	P21 P8 P12
6	P23 P13 P23	P23 P21 P29	P23 P23 P22	P23 P29 P25	P23 P22 P26	P23 P25 P28	P23 P26 P38	P23 P28 P33	P23 P38 P44	P23 P33 P5	P23 P44 P7	P23 P5 P8	P23 P7 P10	P23 P8 P12
7	P29 P13 P23	P29 P21 P29	P29 P23 P22	P29 P29 P25	P29 P22 P26	P29 P25 P28	P29 P26 P38	P29 P28 P33	P29 P38 P44	P29 P33 P5	P29 P44 P7	P29 P5 P8	P29 P7 P10	P29 P8 P12
8	P22 P13 P23	P22 P21 P29	P22 P23 P22	P22 P29 P25	P22 P22 P26	P22 P25 P28	P22 P26 P38	P22 P28 P33	P22 P38 P44	P22 P33 P5	P22 P44 P7	P22 P5 P8	P22 P7 P10	P22 P8 P12
9	P25 P13 P23	P25 P21 P29	P25 P23 P22	P25 P29 P25	P25 P22 P26	P25 P25 P28	P25 P26 P38	P25 P28 P33	P25 P38 P44	P25 P33 P5	P25 P44 P7	P25 P5 P8	P25 P7 P10	P25 P8 P12
10	P26 P13 P23	P26 P21 P29	P26 P23 P22	P26 P29 P25	P26 P22 P26	P26 P25 P28	P26 P26 P38	P26 P28 P33	P26 P38 P44	P26 P33 P5	P26 P44 P7	P26 P5 P8	P26 P7 P10	P26 P8 P12
11	P28 P13 P23	P28 P21 P29	P28 P23 P22	P28 P29 P25	P28 P22 P26	P28 P25 P28	P28 P26 P38	P28 P28 P33	P28 P38 P44	P28 P33 P5	P28 P44 P7	P28 P5 P8	P28 P7 P10	P28 P8 P12
12	P38 P13 P23	P38 P21 P29	P38 P23 P22	P38 P29 P25	P38 P22 P26	P38 P25 P28	P38 P26 P38	P38 P28 P33	P38 P38 P44	P38 P33 P5	P38 P44 P7	P38 P5 P8	P38 P7 P10	P38 P8 P12
13	P33 P13 P23	P33 P21 P29	P33 P23 P22	P33 P29 P25	P33 P22 P26	P33 P25 P28	P33 P26 P38	P33 P28 P33	P33 P38 P44	P33 P33 P5	P33 P44 P7	P33 P5 P8	P33 P7 P10	P33 P8 P12
14	P44 P13 P23	P44 P21 P29	P44 P23 P22	P44 P29 P25	P44 P22 P26	P44 P25 P28	P44 P26 P38	P44 P28 P33	P44 P38 P44	P44 P33 P5	P44 P44 P7	P44 P5 P8	P44 P7 P10	P44 P8 P12

Scheme S2. The 588 single, binary, and ternary combinations made of the 27 selected compounds and catecholamines. Precursor solutions of these 588 combinations were printed inside one well of a standard 4-well-plate using the non-contact liquid dispenser. Stability of the nanocoatings was investigated by immersion of the coated substrate in ethanol for 24 h. Then the substrate was immersed in water and was tilted to allow water droplets to be formed on the coated spots. After treatment with ethanol the combinations that led to formation of water droplets with a diameter of more than 0.55 mm were color coded in dark red, droplets with a diameter between 0.15 mm to 0.55 mm in light red, and droplets with diameter below 0.15 mm were not color coded.

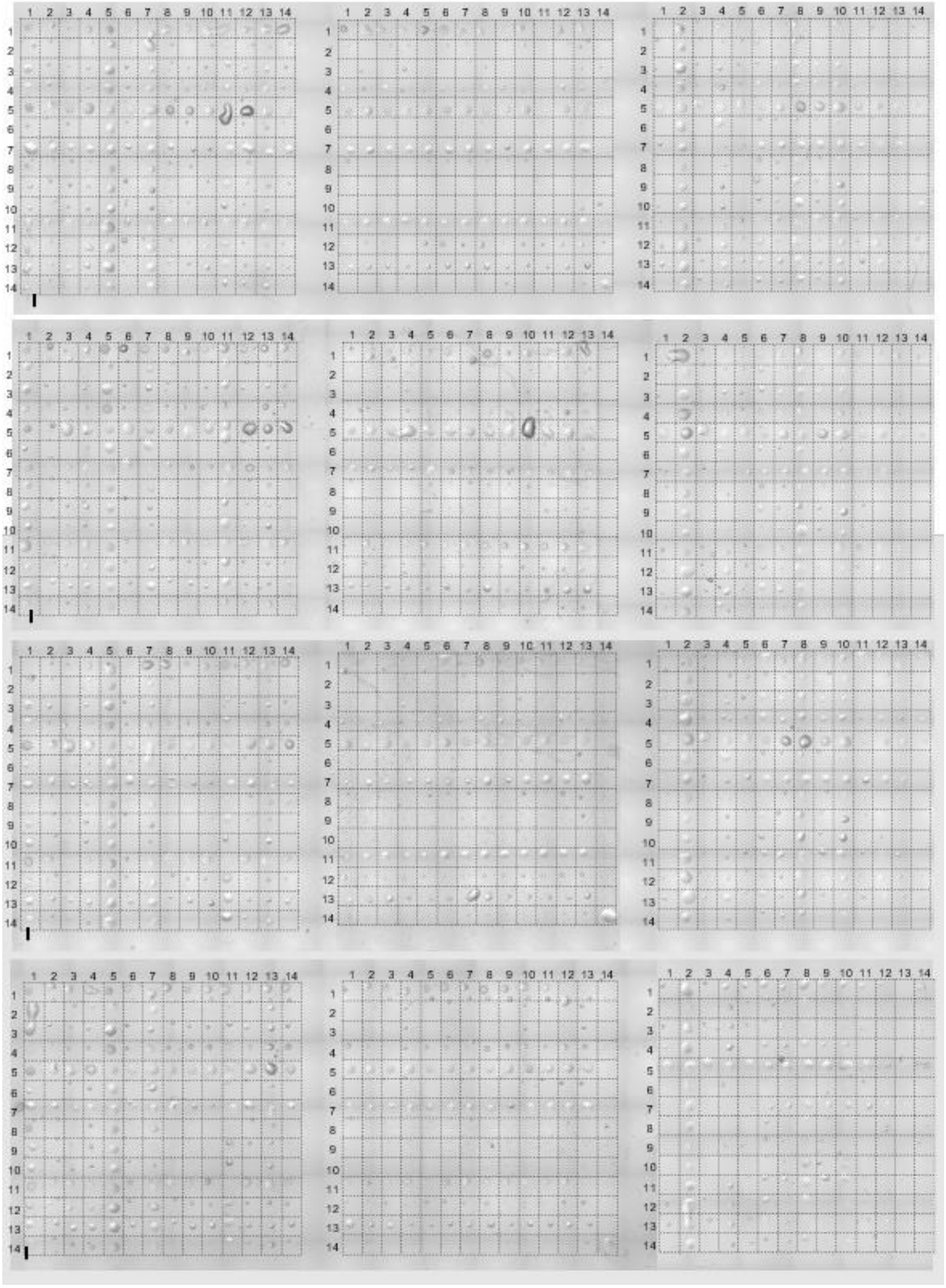

Figure S 19. Spontaneous formation of water droplet microarray (DMA) on substrates treated with precursor solution of the 588 combinations after 24 h in the air (not immersed in ethanol) (four times repetition). Photos are taken by Modellreihe BZ-9000 and merged in Photoshop in each experiment. Scale bar 1mm.

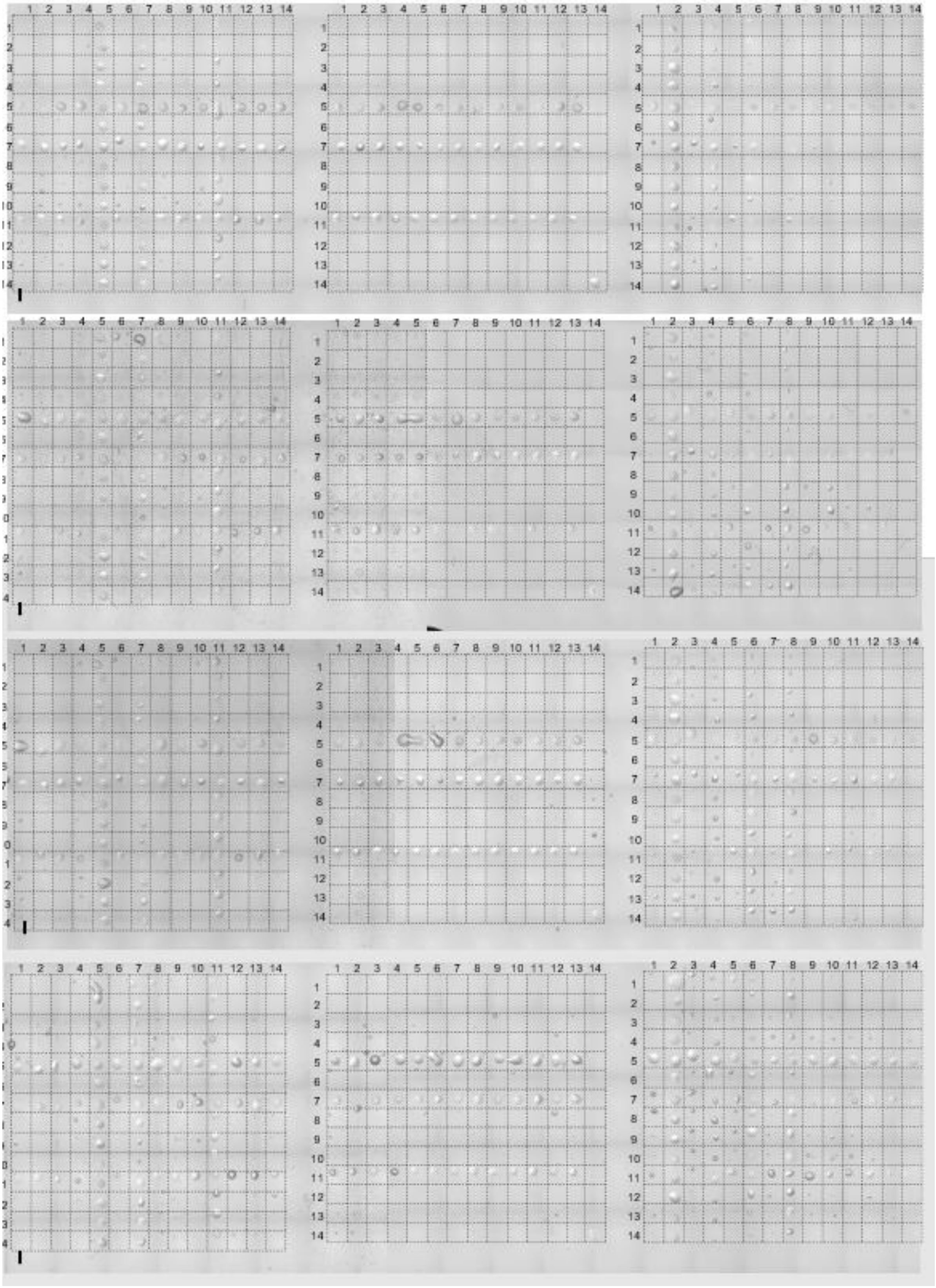

Figure S 20. Spontaneous formation of water droplet microarray (DMA) on substrates treated with a precursor solution of the 588 combinations after 24 h immersion in ethanol (four times repetition). Photos are taken by Modellreihe BZ-9000 and merged in Photoshop in each experiment. Scale bar 1mm.

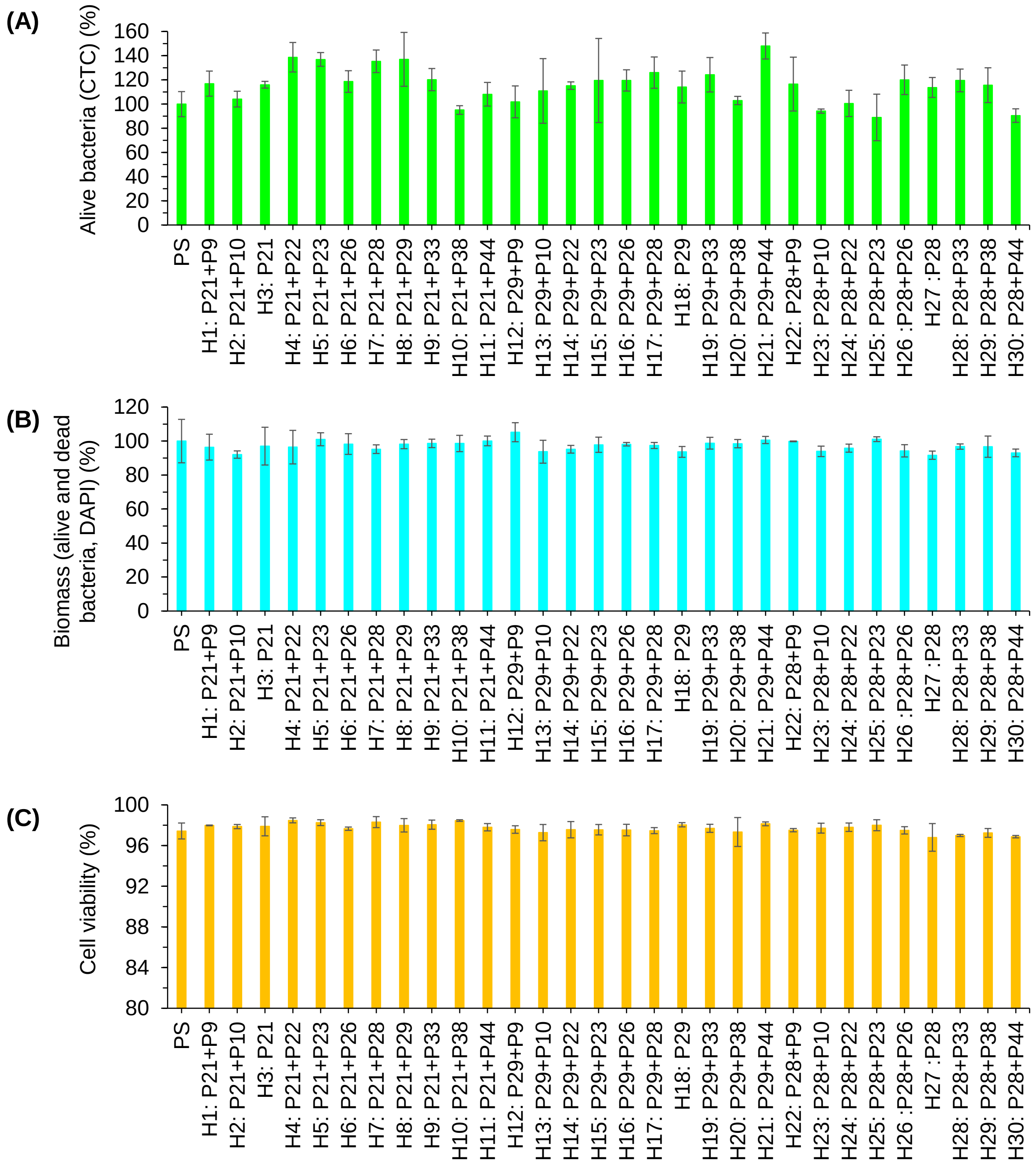

Figure S 21. Bioactivity of the stable nanocoatings derived from hits (H1-H30). (A) Survival rates of *P. aeruginosa* PA 30 strain bacteria after dying with CTC (active bacterial) is shown in the graph. (B) Biomass (alive and dead bacteria after dying with DAPI) is shown in the graph. (C) Cell viability of HeLa cells on the surface coated with the hit nanocoatings. HeLa cells were seeded into pre-coated 96-well plates (1.0×10^4 cells/well) and incubated for 24 h. After that, cells were stained with Hoechst 33342 to visualize cell nuclei and propidum iodide (PI) to distinguish dead cells. Fluorescence images were taken by Olympus IX81 inverted motorized microscope. The number of Hoechst 33342- and PI-positive cells were counted using ImageJ. The cell viability was calculated using the following equation: cell viability (%) = (1- PI-positive cell numbers/Hoechst 33342 positive cell numbers) × 100. Data were presented as mean ± SD of three biological experiments with three technical repeats each time. The statistical significance of the cell viability data was determined with a two-tailed Student t test (P value < 0.05, data not shown since there was no significant differences).

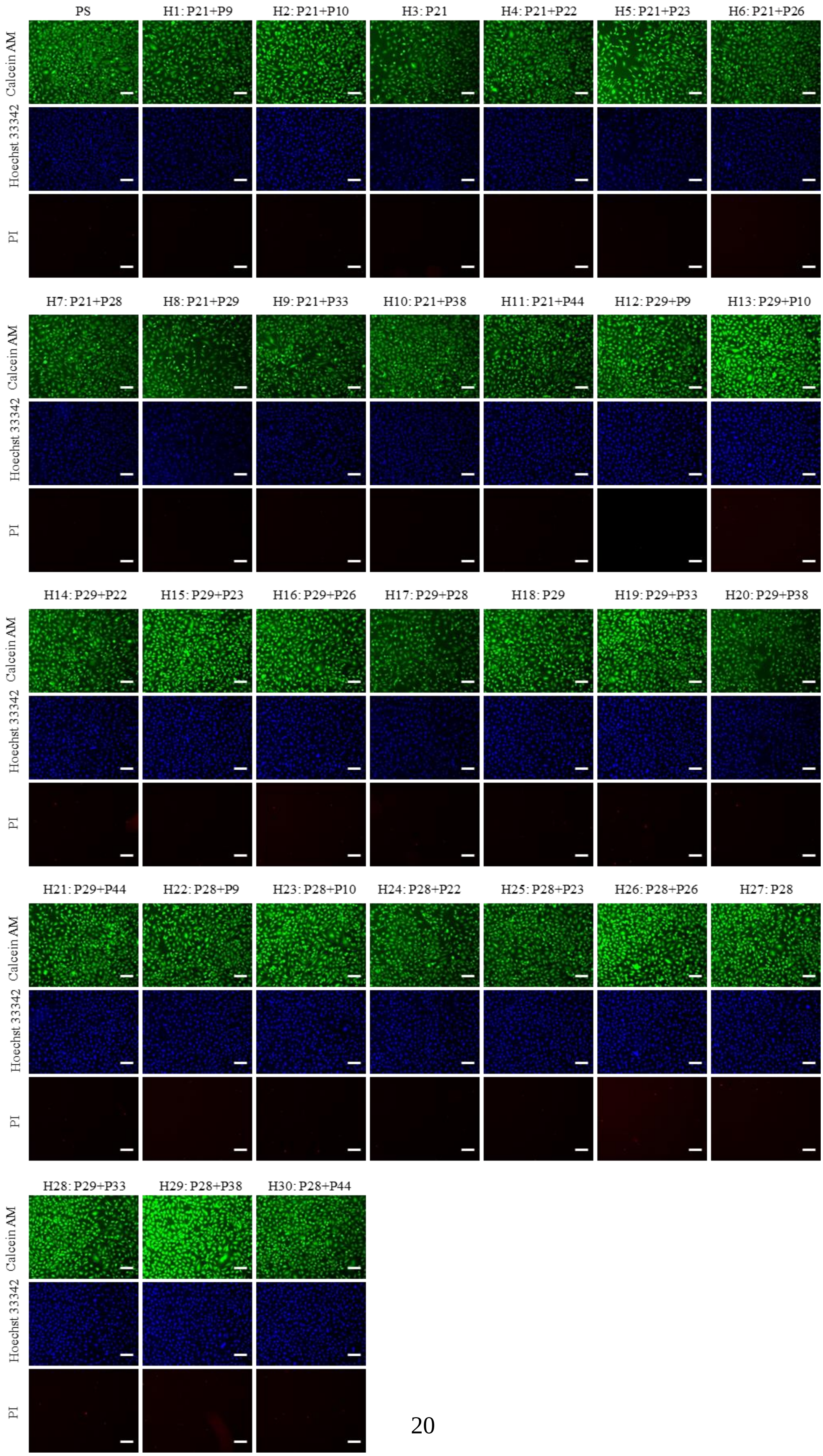
PS
H1: P21+P9
H2: P21+P10
H3: P21
H4: P21+P22
H5: P21+P23
H6: P21+P26
Calcein AM
Hoechst 33342
PI
H7: P21+P28
H8: P21+P29
H9: P21+P33
H10: P21+P38
H11: P21+P44
H12: P29+P9
H13: P29+P10
H14: P29+P22
H15: P29+P23
H16: P29+P26
H17: P29+P28
H18: P29
H19: P29+P33
H20: P29+P38
H21: P29+P44
H22: P28+P9
H23: P28+P10
H24: P28+P22
H25: P28+P23
H26: P28+P26
H27: P28
H28: P29+P33
H29: P28+P38
H30: P28+P44

Figure S 22. Figure S21. Representative fluorescence microscopy images of HeLa cells incubated for 24 h on the surface treated with different hit combinations. Green fluorescence: live cells (Calcein AM). Blue fluorescence: all the cell nuclei (Hoechst 33342). Red fluorescence: dead cells (Propidium iodide (PI)). Scale bar: 100 μm.

Machine learning (ML):

In order to evaluate whether systematic statistical data analysis by means of machine learning (ML) reveals underlying patterns, *e.g.* correlations between chemical structure of the precursor materials and final layer thickness, we trained simple ML models such as random forests based on various representations of the chemical structure of the precursors on the 140 data points shown in Figure 3.

We used a series of different descriptors to represent the chemical structure of the starting materials, as well as a series of different ML models. In particular, we used three different sets of chemical properties *i.e.* descriptors to represent the molecules: Firstly, chemical properties of each molecule calculated using the python package RdKIT; Secondly, similarly to low dimensional fingerprints, the occurrence and number of selected chemical groups (e.g. OH groups); Thirdly, descriptors counting the co-occurrence of chemical groups in both starting materials (e.g. a OH group in one precursor and a NH_2 group in the other). The individual features/descriptors are listed in the SI and defined in a disjoint way, *i.e.* if one of the fingerprint features is a subgraph of another feature, only the largest subgraph present in a molecule is counted.

Due to the low amount of available data, we did not only perform one train-test split, but all results shown in the manuscript are averaged over 10 random train-test splits. In detail, for each train-test split, the models were trained on the respective training data, the performance measure r^2 was computed using the test data in each split, and the overall r^2 score was computed using an average of the r^2 scores on the test data in each train test split. To account for the symmetry between the constituents of a given binary combination, we symmetrised the dataset, so that for each datapoint there was another datapoint just with the feature vectors of the two molecules exchanged. This step took place after splitting the data in training and test sets, to prevent leaking of knowledge about the test set into the train set. This step slightly improved the r^2 score and reduced the variance of most of the models.

The best machine learning models that we trained were able to predict the experimentally observed layer thickness of a test set with a correlation coefficient between predicted and measured thicknesses of r^2 ~ 0.3. This is significantly above a random guess (r^2 = 0.0), but still very low compared to highly accurate machine learning models reaching r^2 values of close to 1.0. This suggests that either the amount of data is not sufficient to train a model that can generalize reliably to new molecules, or the chemical descriptors we used do not include enough information to predict the thicknesses (a more complete representation in form of *e.g.* extended connectivity fingerprints was not possible due to the small amount of datapoints and the resulting overfitting of the models), or the data contains intrinsic noise.

Therefore, we mainly focused our analysis on the question which features/descriptors are most relevant to explain the experimentally observed layer thickness. The main result is that the number of OH groups and the octanol-water partition coefficient *logP* are the most important descriptors. The best results we obtained using the chemical properties and a Random Forest Regression model were $r^2 = 0.30 \pm 0.16$, where the *logP* attribute had an importance of 0.170, *i.e.* 17.0% of the variation in thickness of the combination of two molecules could be explained by their variation in the *logP*. For the tests using chemical fingerprints as features, the number of OH groups was the by far most important feature and accounts for about 30% of the variation of the thickness. Further relevant chemical groups are 3C2OH (exactly two OH groups in a ring with one carbon atom distance, e.g. 1,3-dihydroxybenzene) and COOH (carboxylic acid, e.g. 3,4-Dihydroxybenzoic Acid).

However, none of the features showed a clear correlation with the thickness. Correlation plots of the two identified most important features (number of OH groups and logP) can be seen in Figure S18. Our linear regression models performed even worse than the non-linear ones, indicating that there is no linear correlation between single input descriptors (e.g. the number of OH groups or logP) and the coating thickness. Only non-linear combinations of input descriptors (in form of Random Forest models or Gaussian Process models) improved the prediction accuracy slightly.

Because the direct prediction of layer thicknesses turned out to be hard, we trained models to predict the scalar product between the rows/columns in Figure 3, which quantifies the similarity between two molecules when reacting with a series of reference molecules. Overall, the best performance was obtained using Random Forest Regression and Gaussian Process models. The performance of the Random Forest went up to $r^2 = 0.33 \pm 0.11$ which is a small improvement in comparison to the result discussed above. However, the prediction of similarity is less interpretable than the direct prediction of the layer thickness. Repeating the feature importance analysis confirmed the results from above: The *logP* still is the most

important feature with an importance of 0.12. For the tests using chemical fingerprints as input representation, the most important feature still was the number of OH groups, still with an importance of about 30%.

The following representations were used to train ML models:

1. Chemical properties of molecules computed with RdKIT: BalabanJ, BertzCT, ExactMolWt, FpDensityMorgan1, FpDensityMorgan2, FpDensityMorgan3, FractionCSP3, HallKierAlpha, HeavyAtomCount, HeavyAtomMolWt, Ipc, Kappa1, Kappa2, Kappa3, LabuteASA, MaxAbsEStateIndex, MaxAbsPartialCharge, MaxEStateIndex, MaxPartialCharge, MinAbsEStateIndex, MinAbsPartialCharge, MinEStateIndex, MinPartialCharge, MolLogP, MolMR, MolWt, NHOHCount, NOCount, NumAliphaticCarbocycles, NumAliphaticHeterocycles, NumAliphaticRings, NumAromaticCarbocycles, NumAromaticHeterocycles, NumAromaticRings, NumHAcceptors, NumHDonors, NumHeteroatoms, NumRadicalElectrons, NumRotatableBonds, NumSaturatedCarbocycles, NumSaturatedHeterocycles, NumSaturatedRings, NumValenceElectrons, RingCount, TPSA, qed (more information can be found in the RdKIT documentation)
2. Fingerprint vectors describing the number of occurences of the following chemical groups in the molecules: OH (hydroxyl), C=O (carbonyl), NH_2 (amine on an aromatic cycle), COOH (carboxylic acid), 3C2OH (two hydroxyl groups with one C atom distance, meta position), 2COH (two hydroxyl groups directly adjacent, ortho position), 3COH (three adjacent hydroxyl groups), CNH_2 (amine at the end of an aliphatic chain)
3. Fingerprint vectors describing combinations of the chemical groups described in (2).

We trained the following ML models: Linear Regression, Random Forest Regression, Gradient Boosting Regression, Neural Networks, Gaussian Process Regression. For the Random Forest and Gradient Boosting models, we extracted the feature importances. The best performance on the tasks described in the main text using the different types of input representations described above was achieved using the Random Forest model, followed by the Gaussian Process model. Detailed results of selected tests are listed below.

Task 1: Prediction of thickness

Best model: Random Forest, trained on the 10 most relevant chemical RdKIT features for each molecule (i.e. 20 features in total)

(determined using a Random Forest model trained on all features). Performance: r^2 = 0.30 ± 0.16. Most important features (relative feature importance): MolLogP (0.170), BalabanJ (0.119), MinAbsEStateIndex (0.103), FpDensityMorgan3 (0.097), qed (0.093)

The best model with fingerprint vectors as features was the Random Forest. Performance: r^2 = 0.16 ± 0.12. Most important features (relative feature importance): OH (0.326), 3C2OH (0.109), COOH (0.109), 2COH (0.108), 3COH (0.104)

Task 2: Prediction of scalar products (similarities)

Best model: Random Forest, trained on the 10 most relevant chemical RdKIT features for each molecule (i.e. 20 features in total)

(determined using a Random Forest model trained on all features). Performance: r^2 = 0.33 ± 0.11. Most important features (relative feature importance): MolLogP (0.120), MinAbsEStateIndex (0.117), MinEStateIndex (0.115), qed (0.110), FpDensityMorgan1 (0.099)
